# Evidence that avian reovirus σNS is an RNA chaperone: implications for genome segment assortment

**DOI:** 10.1093/nar/gkv639

**Published:** 2015-06-24

**Authors:** Alexander Borodavka, James Ault, Peter G. Stockley, Roman Tuma

**Affiliations:** School of Molecular and Cellular Biology & Astbury Centre for Structural Molecular Biology, University of Leeds, Leeds, LS2 9JT, UK

## Abstract

Reoviruses are important human, animal and plant pathogens having 10–12 segments of double-stranded genomic RNA. The mechanisms controlling the assortment and packaging of genomic segments in these viruses, remain poorly understood. RNA–protein and RNA–RNA interactions between viral genomic segment precursors have been implicated in the process. While non-structural viral RNA-binding proteins, such as avian reovirus σNS, are essential for virus replication, the mechanism by which they assist packaging is unclear. Here we demonstrate that σNS assembles into stable elongated hexamers *in vitro*, which bind single-stranded nucleic acids with high affinity, but little sequence specificity. Using ensemble and single molecule fluorescence spectroscopy, we show that σNS also binds to a partially double-stranded RNA, resulting in gradual helix unwinding. The hexamer can bind multiple RNA molecules and exhibits strand-annealing activity, thus mediating conversion of metastable, intramolecular stem-loops into more stable heteroduplexes. We demonstrate that the ARV σNS acts as an RNA chaperone facilitating specific RNA–RNA interactions between genomic precursors during segment assortment and packaging.

## INTRODUCTION

Avian reoviruses (ARVs) are commercially significant pathogens that cause considerable losses in the poultry industry worldwide (1). ARVs belong to the genus *Orthoreovirus* of the *Reoviridae* family, which encompasses numerous human and animal pathogens, including rotaviruses, bluetongue virus and coltiviruses. Reoviruses possess double-stranded (ds) RNA genomes, partitioned into 10–12 genomic segments (2). The ARV genome (23.5 kb) is composed of 10 dsRNA segments, encoding 8 structural and at least four nonstructural proteins (1). The genomic dsRNA segments are encased within two concentric protein shells, forming the outer capsid and the core. Apart from genomic segments the core contains multiple copies of the RNA-dependent RNA polymerase and the capping enzyme (2). Upon infection the outer capsid is proteolytically removed, resulting in the transcriptional activation of the core. The released core extrudes mRNAs into cytoplasm (3), where they are used for both translation and as genome segment precursors.

RNA replication and morphogenesis of reoviruses occurs exclusively within cytoplasmic inclusion bodies, also known as viral factories, or ‘viroplasms’ (4). ARV viroplasms are primarily made of the nonstructural protein μNS in association with another nonstructural protein σNS (3,5,6). The mechanism of segment assortment, by which exactly one of each of the multiple genomic precursors is selected and packaged into individual virions, is largely unknown, remaining one of the most significant questions in the biology of pathogenic dsRNA viruses. Previous studies of other members of the *Reoviridae* family suggest that multiple RNA–RNA interactions are involved in segment assortment (7–9). These interactions are believed to drive formation of a hypothetical ‘assortment complex’, which includes a full set of genome segment precursors, single-stranded (ss) RNAs, destined for encapsidation (10–12). In ARVs and mammalian reoviruses (MRVs) nonstructural RNA-binding proteins σNS appear to be important in this process, although their functions remain poorly understood (3,13,14).

While σNS proteins from ARVs and MRVs share very little sequence similarity, both proteins are known to form oligomers that bind ssRNA, as well as ssDNA, having very low affinity for double-stranded nucleic acids (6,13,14). Here, using a combination of various biophysical techniques, we demonstrate that the ARV σNS assembles into elongated hexamers, capable of binding strands or segments of ssRNA with nanomolar affinity. Hexamer binding causes local destabilization (unwinding) of RNA secondary structure, resulting in formation of ribonucleoprotein complexes of variable stoichiometry. Upon helix unwinding, σNS promotes annealing of complementary strands, yielding more stable intermolecular duplexes with extended complementarities. Our results show that the ARV σNS is capable of accelerating RNA folding, thus functioning as an RNA chaperone (15), consistent with its role in genomic segment precursor selection by facilitating specific RNA–RNA interactions in viroplasms.

## MATERIALS AND METHODS

### Plasmid construction, protein expression and purification

Total RNA, extracted from chicken embryo fibroblast cells infected with the ARV strain 1733, was a gift from Prof. Javier Benavente (University of Santiago de Compostela). The RNA was reverse-transcribed using Superscript III Reverse Transcriptase and random hexamer oligonucleotide primers (Invitrogen). Oligonucleotide primers F_sns and R_sns (Supplementary Table S1) were used to PCR-amplify the resulting cDNA, with NdeI and XhoI restriction sites used for ligating the resulting double-digested σNS-coding DNA fragment into a linearized pET-15b vector (Novagen). The resulting pET-15b-σNS DNA construct was verified by sequencing.

pET-15b-σNS-transformed BL21(DE3)pLysS *Escherichia coli* cell cultures grown at 37°C were induced with 1 mM isopropyl-β-d-thiogalactopyranoside (IPTG) upon reaching optical density (600 nm) of 0.6–0.7, after which the protein expression was continued at 21°C overnight. Following a low-speed centrifugation harvesting, cells were resuspended and incubated for 30 min in lysis buffer (50 mM Tris–HCl, pH 8, 200 mM NaCl, 1% Tween 20, 0.2 mg/ml chicken egg lysozyme), complemented with a complete protease inhibitor cocktail tablet (Roche), and then treated with DNAseI (0.1 mg/ml) for 15 min at room temperature (RT) before cellular debris were removed by centrifugation at 20 000r pm for 30 min. The clarified cell lysate was loaded onto a pre-equilibrated Ni^2+^-charged HisTrap FF column (GE Healthcare), washed with buffer A (1.2 M NaCl, 50 mM Tris–HCl pH 8, 40 mM imidazole and 0.5% Tween 20), and eluted using a linear gradient of buffer B (0.2 M NaCl, 50 mM Tris–HCl pH 8, 1 M imidazole). Collected protein-containing fractions were dialysed against buffer C (50 mM Tris–HCl pH 8, 1 mM EDTA, 200 mM NaCl) at 4°C, treated with human plasma thrombin (1 U/100 μg of recombinant protein) for 12 h at 22°C in order to remove His-tags. Protein samples were then loaded onto a pre-equilibrated HiTrap Q column, washed with buffer D (50 mM Tris–HCl pH 8, 1 mM EDTA, 50 mM NaCl), and the protein was eluted with buffer E (50 mM Tris–HCl pH 8, 1 M NaCl). Concentrated σNS samples were injected onto a Superdex 200 10 × 300 GL column (GE Healthcare) in 25 mM HEPES–Na, pH 7.5, 150 mM NaCl. After the final purification step, the *A*_260_/*A*_280_ absorbance ratio of ∼0.57 suggested negligible contamination with nucleic acids.

For strand-annealing assays, rotaviral protein NSP2 was expressed and purified, as previously described in (16).

### RNA and DNA oligonucleotides used for σNS binding assays

All oligonucleotides were obtained from Integrated DNA Technologies (IDT). A 120-nt long RNA sequence was designed with a fixed nucleotide composition distribution (A = 5%, C = 40%, G = 5%, T = 50%) in order to minimise any potential base-pairing. The resulting nucleotide sequence was examined using the mfold web server (17), and the RNA sequence with the lowest propensity to form intramolecular base pairs was chosen. A DNA ultramer (Supplementary Table S1) was used for transcribing a 120-nt long RNA. This transcript, as well as longer RNA transcripts (3569-nt long bacteriophage MS2 RNA) were fluorescently labelled with AlexaFluor 488 (AF488) dye (Life Technologies), as described in (18). A 30-nt and a 20-nt long RNAs, representing portions of a 120-nt long RNA, were chemically synthesized, 3′-end labelled (AF488) and PAGE-purified. A 46-nt long DNA oligonucleotide (Supplementary Table S1) was designed to form a hairpin structure and was synthesized with fluorescent labels at both the 5′ and 3′ ends (AF488 and Texas Red) (IDT).

### Sedimentation velocity (SV) analytical ultracentrifugation

Sedimentation velocity data were collected from samples, loaded into 1.2-cm path length, 2-sector meniscus-matching epon centrepiece cells in an An50-Ti rotor using Optima XL-I Beckman Analytical Ultracentrifuge (Beckman Instruments, Palo Alto, CA, USA). SV analyses were conducted at 21°C with protein concentrations ranging between 5 and 25 μM and RNA concentrations of 50–60 nM. Changes in solute concentration were detected using absorbance scans at 260 nm for nucleic acids and 280 nm for proteins, as well as by interference. Each cell was scanned 200 times during the 12 h run, preceded with 1 h pre-run period in the rotor at a given speed (30 000 and 48 000 rpm, respectively). Buffer density and viscosity were calculated using Sednterp (19). Partial specific volume values for hydrated RNA were assumed 0.53–0.55 cm^3^/g (20), and 0.7321 cm^3^/g for σNS (calculated from its amino acid composition) with an estimated hydration level of 0.3733 g/g (19).

Radial absorbance profiles were fitted to a continuous distribution c(S) Lamm equation model using the Sedfit version 12.1b software (21). During the analysis of the sedimentation velocity profiles systematic time-invariant and radial-invariant noise components were calculated and taken into account. Models for single and multiple discrete species were used, and the location of the meniscus was treated as a floating parameter. The resulting meniscus position was then used in the c(S) distribution analysis. Maximum entropy regularization was used to calculate the size distribution within a confidence level of 0.68 of the best-fit distribution (22). Equivalent Stokes radii (R*_h_*) were calculated using Einstein–Stokes relationship, implemented in Sedfit (21).

### Sedimentation equilibrium (SE) analytical ultracentrifugation

Protein samples (6–25 μM) were examined by SE, which was attained at 8000 rpm for 24 h and 12 000 rpm for 12 h at 20°C in an eight-hole An50 Ti rotor. Global analysis of several datasets of radial absorbance obtained at different loading concentrations and rotor speeds was performed in Sedphat (23), and SE data were fit to a single ideal species model (24) in order to estimate apparent molecular masses.

### Negative staining transmission electron microscopy (TEM)

Five microliters of the σNS–RNA complex, used in SV experiments (see above), was applied onto carbon-coated grids (Agar Scientific) to absorb for 1 min, after which the excess of sample was removed by blotting with filter paper. Grids were stained with 2% (w/v) uranyl acetate, and examined using JEOL 1200EX transmission microscope operating at 80 kV at 30 000× and 40 000× magnifications.

### Circular dichroism (CD) spectroscopy

Protein samples (4–32 μM in 100 mM NaF, pH 7.8) were analysed in a 0.5-mm path length cuvette using a Chirascan CD spectrometer (Applied Photophysics). CD spectra were acquired between 260 and 170 nm at 21°C. Two spectra were recorded and averaged for each concentration, followed by subtraction of the buffer spectrum. ProData Viewer software (Applied Photophysics) was used to view the recorded spectra and to assess the quality of the spectral data by examining HT voltage (absorbance). Measured ellipticities (mdeg) were expressed in terms of mean residue ellipticity (deg cm^2^/dmol). CD spectra were deconvolved by applying CDSSTR algorithm (25) using SP175 reference database (26,27), implemented in CD analysis suite DICHROWEB (28).

CD spectra of RNA were acquired in the 240–320 nm spectral range using a 1 cm-long path cell, thermostated at 37°C, as described in (29). CD spectra were recorded for the MS2 phage RNA (∼3.6 kb, 0.2 mg/ml), before and after incubation with 5–20 μM σNS for 15 min. RNA secondary structure transitions upon thermal melting were also monitored at various temperatures up to 95°C.

### Dynamic light scattering (DLS)

Apparent hydrodynamic radii of σNS species (10–60 μM) were measured by DLS using a PrecisionDeconvolve PDDLS/Batch platform, equipped with a PD2000 DLS detector. All measurements were performed in a buffer with an estimated viscosity, *η* = 1.002 mPa s, and density, *ρ* = 1.00453 g/cm^3^ at 25°C. Hydrodynamic radius distributions were computed using Precision Deconvolve regularization-based software (Precision Detectors).

### Non-covalent electrospray ionisation ion mobility-mass spectrometry (ESI-IMS-MS)

Mass spectra of protein samples (12–150 μM in 200 mM ammonium acetate, pH 7.8) were acquired in the positive-ion mode using a Synapt HDMS (Waters, UK), with quadrupole-orthogonal acceleration time-of­-flight geometry and a built-in traveling wave ion mobility device. Protein mass spectra were also acquired using the LCT Premier (Waters, UK) specially modified for the analysis of non-­covalently bound macromolecular complexes. The ESI conditions were optimized for the highest sensitivity detection of multimeric complexes in the gas phase (30). The drift times for the IMS data have been extracted using MassLynx software and the *m/z* spectra were plotted against the drift time using Driftscope software (Waters, UK). Calibration of the drift time cross-section was done by the combined analyses of denatured proteins (equine cytochrome *c*, β-lactoglobulin, avidin, alcohol dehydrogenase, pyruvate kinase). For each protein, the individual charge state ions were identified by *m/z* ratios and their measured drift times were plotted against collision cross-sections (Ω), taken from Prof. D. Clemmer's database (Indiana University Bloomington), as described previously (31,32).

### Solution small angle X-ray scattering (SAXS)

Experimental SAXS datasets from solutions were collected at the X33 beam line (EMBL, DESY, Hamburg). SAXS data were acquired at 21°C using a 2D Photon counting Pilatus 1M-W pixel X-ray detector with a sample to detector distance set to 2.7 m. Protein samples (1.1 – 9.5 mg/ml in 50 mM Tris–HCl, pH 7.8, 100 mM NaCl) were examined, while serial dilutions (1–10 mg/ml) of bovine serum albumin (BSA) in the same buffer were used for calibrating *I*_0_ values. Data were processed using ATSAS v. 2.5.1 (33,34). Background subtraction and data quality checks were performed in PRIMUS (35). Radii of gyration (*R*_g_) were estimated using AUTORG (33). Scattering curves that were collected at several concentrations were scaled and merged using PRIMUS package. The resulting scattering curves served as input for *ab initio* low resolution shape reconstruction algorithms DAMMIN and DAMMIF (36). Multiple models with different assumed symmetries were generated (225 in total), and their ion collisional cross-sections were estimated using Leeds algorithm (37). Models with computed cross-sections within 10% (estimated experimental error) of the value measured by IMS-MS were selected (∼70% of all models generated), aligned, selected and superimposed with DAMSEL and DAMSUP and finally averaged with DAMAVER (38). The resulting model was iteratively filtered (DAMFILT) to the volume with a computed cross-sectional area, corresponding to the hexamer as measured by IMS-MS.

### Fluorescence correlation spectroscopy (FCS) data collection and analysis

FCS measurements were performed on a custom-built FCS confocal setup (39), as described in (18). FCS data were analyzed by non-linear least-squares fitting with an autocorrelation function model accounting for a single diffusion component and the triplet state dynamics in Matlab (ver 7.11, MathWorks). *R_h_* values were estimated based on measured diffusion time values for AF488 dye molecule as described before (18).

### Fluorescence anisotropy (FA) and binding affinity determination

AF488-labelled 120-nt, 30-nt and 20-nt long RNAs were used for estimating binding affinities of σNS to ssRNAs. All measurements were performed at 21°C using a Fluorolog spectrofluorimeter (Horiba Jobin-Yvon). σNS was titrated (10 nM–10 μM final concentrations) into 1 nM of each of the RNA in 10 mM HEPES–NaOH, pH 7.5, 150 mM NaCl, allowing equilibration for 30 min prior FA data collection. Normalized anisotropy was plotted as a function of protein concentration and fitted to a single-site binding model using OriginPro 9.0 software.

### Ensemble and single-molecule FRET helix-unwinding assays

Fluorescently labeled 46-nt long probe with a FRET donor (AF488) and acceptor (Texas Red) pair at the 5′ and 3′ ends, respectively, was designed (Supplementary Table S1). Since σNS was shown to bind ssDNA, as well as ssRNA (14), due to the high cost and low yield, a dual-labeled, 46-nt long ssDNA was synthesized and HPLC purified instead of ssRNA. Ensemble FRET efficiencies were measured for 1 nM probe in the absence of σNS, upon thermal unfolding at 80°C, and in presence of the increasing amounts of σNS (50 nM–10 μM) in the assay buffer (10 mM HEPES–NaOH, pH 7.5, 150 mM NaCl). Fluorescence intensities at 515 nm (donor) and 615 nm (acceptor) were recorded upon excitation with 488 nm, and FRET efficiencies were estimated as described in (40). For single-molecule FRET measurements a custom-made inverted confocal microscope setup equipped with Alternating Laser Excitation (ALEX) was used (41), as previously described in (42). Dual-labeled 46-nt long probe (10 pM) was measured in assay buffer with variable amounts of σNS (25 nM–20 μM) at 37°C. During data acquisition laser alternation period was set to 100 μs with 488 nm laser intensity set to 90 μW and the 594 nm laser intensity set to 60 μW. Burst selection was performed with a 10 kHz threshold, with the burst photon number set to 200 photons. Uncorrected ratiometric observables E and S were calculated after burst identification as described in (42).

### RNA strand-annealing assay

Two 33-nt long oligonucleotides, 33A and 33B, with self-complementary stem-forming regions (Supplementary Table S1) were designed with the aid of mfold (17) and used in the assay. Each 33A and 33B (100 μM) were separately heat-annealed for 5 min at 85°C in 100 mM NaCl, 10 mM MgCl_2_, 50 mM Tris–HCl, pH 7.9, slowly cooled and diluted in assay buffer (25 mM HEPES–Na, pH 7.5, 0.05% Tween 20, 150 mM NaCl, 2 mM DTT) to 5 ng/μl. Reactions were set up with equimolar amounts of 33A and 33B (200 nM total concentration) and variable amounts (1–20 μM) of the ARV σNS or acetylated BSA (negative control). Reactions were allowed to proceed at 37°C for 5–30 min before they were stopped by adding gel-loading buffer, after which they were kept on ice before resolving products on a 1× TBE 15% PAGE. Gels were stained with SYBR Gold (Invitrogen) and visualized by a fluorescence scanner with a 488 nm laser excitation.

Additional strand-annealing assays were performed with viral RNA fragments, encompassing the last 91 nucleotides of the 3′ end (1552–1643 nt) of segment s1 precursor and a similarly sized RNA fragment (422–513 nt) of segment s4 precursor (S1133 strain of ARV). These regions were identified using RactIP tool (43) with the minimum folding energies and structures of the respective RNA sequences computed using mfold (17). DNA Ultramers (IDT), incorporating T7 promoter sequences upstream of either s1 or s4 sequence, were designed, commercially synthesised and used for *in vitro* transcription using T7 polymerase. The resulting RNA products were purified, and each RNA strand was heat-annealed (as described above) prior strand-annealing reactions. For strand-annealing reactions, s1 and s4 RNAs (1.5 μM each) were incubated with 50–70 μM σNS for 5–15 min at 37°C. Similarly, strand-annealing reactions were set up with the rotaviral nonstructural protein NSP2. All reactions were stopped by adding 40 μg of proteinase K (NEB), and incubating for another 15 min. RNA samples were resolved on a native 12% 1× TBE PAGE and visualised by staining with SYBR Gold (Invitrogen).

## RESULTS

### ARV σNS forms stable elongated hexamers

**Figure 1.**
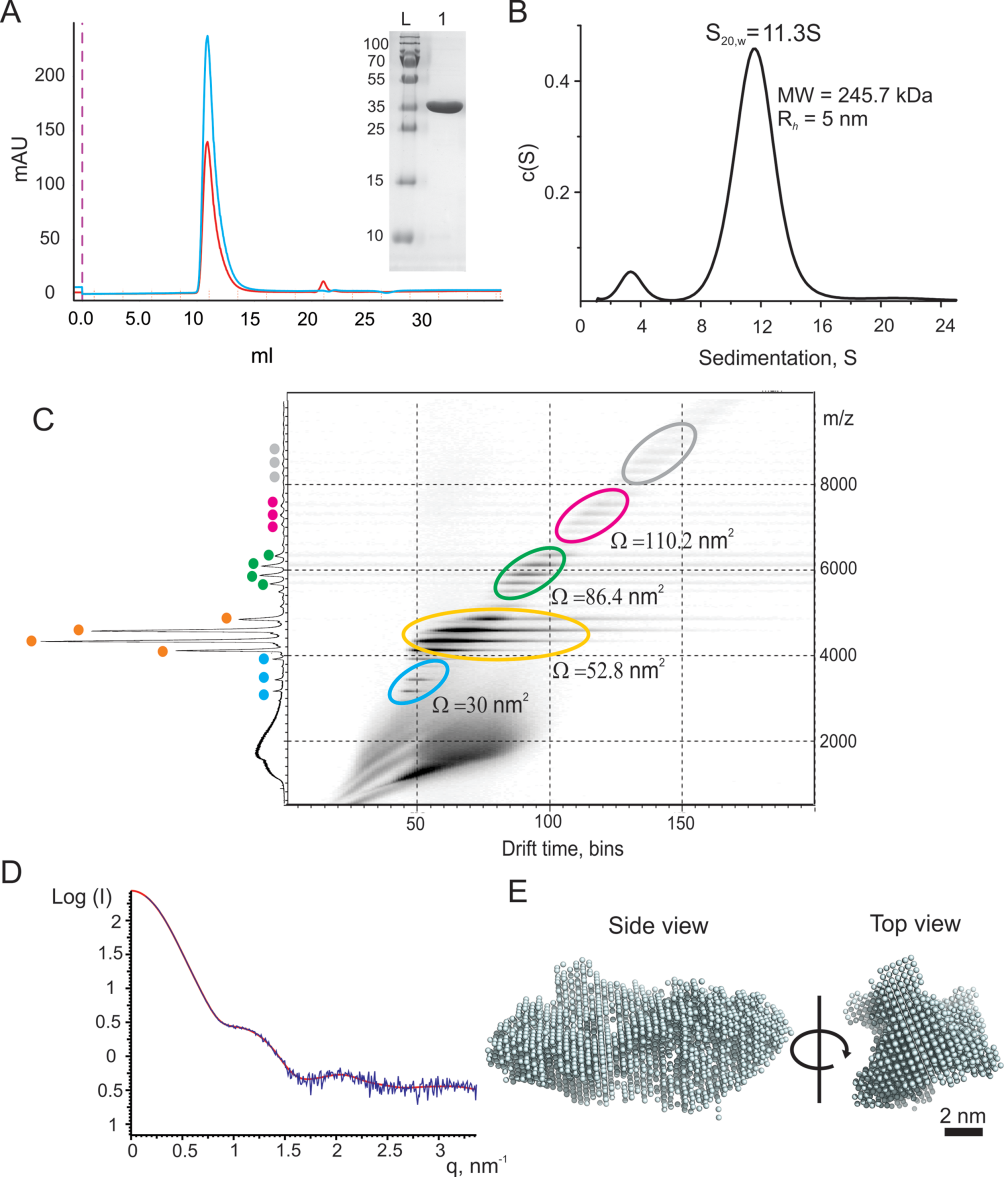
Quaternary structure of the ARV σNS. (**A**) Size exclusion chromatography (Superdex 200 10 × 300GL column) of σNS after His-tag removal and anion-exchange purification. 260 and 280 nm absorbance is shown in red and blue, respectively. Inset: SDS-PAGE of the purified protein (L – molecular weight marker with masses in Da shown on the right). (**B**) Sedimentation velocity analysis of the ARV σNS sedimenting at 11.3 ± 0.3S at 10 μM protein concentration. (**C**) ESI-IMS-MS Driftscope plot of the ARV σNS. Protein monomers and oligomers are highlighted in differently colored ovals (blue – monomers, orange – dimers, green – tetramers, magenta – hexamers, and gray – low intensity higher order oligomers) are separated by their drift times and *m*/*z* values. The cumulative *m*/*z* scan spectrum is shown on the right (y-axis) and the detected charge state ions are labeled according to the color scheme used in the Driftscope plot. Averaged collisional cross-sectional areas (*Ω*) values (in nm^2^) for the lowest detected charge states are shown next to the corresponding protein oligomers. The *m*/*z*, molecular masses, drift time and cross-sectional area values are summarised in Supplementary Table S2. (**D**) Merged and buffer corrected experimental SAXS curves (blue), taken for a range of σNS concentrations (30–150 μM), with overlaid reconstructed fit (red). (**E**) A bead model of the ARV σNS hexamer reconstructed from the SAXS data. Multiple models compatible with SAXS data were produced and filtered according to their estimated collisional cross-sectional areas (*Ω*), as described in ‘Materials and Methods’ section.

A full-length recombinant ARV σNS was expressed and purified as described in ‘Materials and Methods’ section, and a homogeneous, intact protein sample was obtained by size-exclusion chromatography (Figure 1 A and Supplementary Figure S1). Its early elution from the size-exclusion column suggests that σNS oligomerizes, whilst circular dichroism spectroscopy confirms that the protein is folded, and composed of ∼35% β-strands and ∼29% α-helices (Supplementary Figure S1). Sedimentation equilibrium analysis of the protein at micromolar concentrations yields a mass of 259.3 ± 12.0 kDa, suggesting that under these conditions it is a hexamer (Supplementary Figure S2). Sedimentation velocity analysis (Figure 1 B), combined with the apparent hydrodynamic radius (*R_h_*) of 5.0 ± 0.4 nm (estimated by dynamic light scattering, Supplementary Figure S3 A), reveals 11.3S oligomers with a corresponding mass of 237.6 ± 23.0 kDa. Taken together, these analyses demonstrate that at low micromolar concentrations the ARV σNS assembles into stable 11.3S hexamers.

Previously, smaller oligomeric species of the ARV σNS were reported (10). We therefore hypothesized that these smaller oligomers could be assembly intermediates of the hexamer. In order to characterize the assembly of hexamers, we employed native electrospray ionization ion-mobility spectrometry mass-spectrometry (ESI-IMS-MS). As expected, σNS hexamers are observed (Figure 1 C), while the mass-spectrum also contains monomers, dimers and tetramers. These smaller oligomers likely result from dissociation during electrospray ionization, since neither velocity nor equilibrium sedimentation data indicate mass heterogeneity. The prevalence of oligomers with even number of subunits suggests that a dimer is the building block of the hexamer. Since the lower molecular weight species are only detected by the ESI-MS, it is likely that assembly of σNS dimers into hexamers is driven by hydrophobic interactions, which are significantly weaker in the gas phase.

Given the monodisperse nature of the hexamer, we characterized its shape using small-angle X-ray scattering (SAXS) (Figure 1D). The estimated mass and size of a σNS hexamer is in agreement with its hydrodynamic properties, with a radius of gyration (*R_g_*) of 5.3 ± 0.3 nm, while the *R_g_*/*R_h_* ratio > 1 (44) suggests that its shape is elongated. We then performed *ab initio* shape reconstruction using the SAXS data (36), without symmetry (P1, Supplementary Figure S3B), or with P32 symmetry imposed, treating a hexamer as a trimer of dimers. Multiple reconstructed best-fit SAXS models were ranked using their estimated IMS-MS cross-section values (Supplementary Figure S4 and Supporting Table S2), selected, superimposed and averaged (see ‘Materials and Methods’ section for details). The resulting prolate bead model is shown in Figure 1E, consistent with a shape of a hexamer, which assembles as a trimer of dimers at low micromolar protein concentrations. A similar shape is obtained by reconstruction without any imposed symmetry (Supplementary Figure S3 B).

### σNS hexamers bind ssRNAs with high affinity, forming ribonucleoproteins of variable stoichiometry

**Figure 2.**
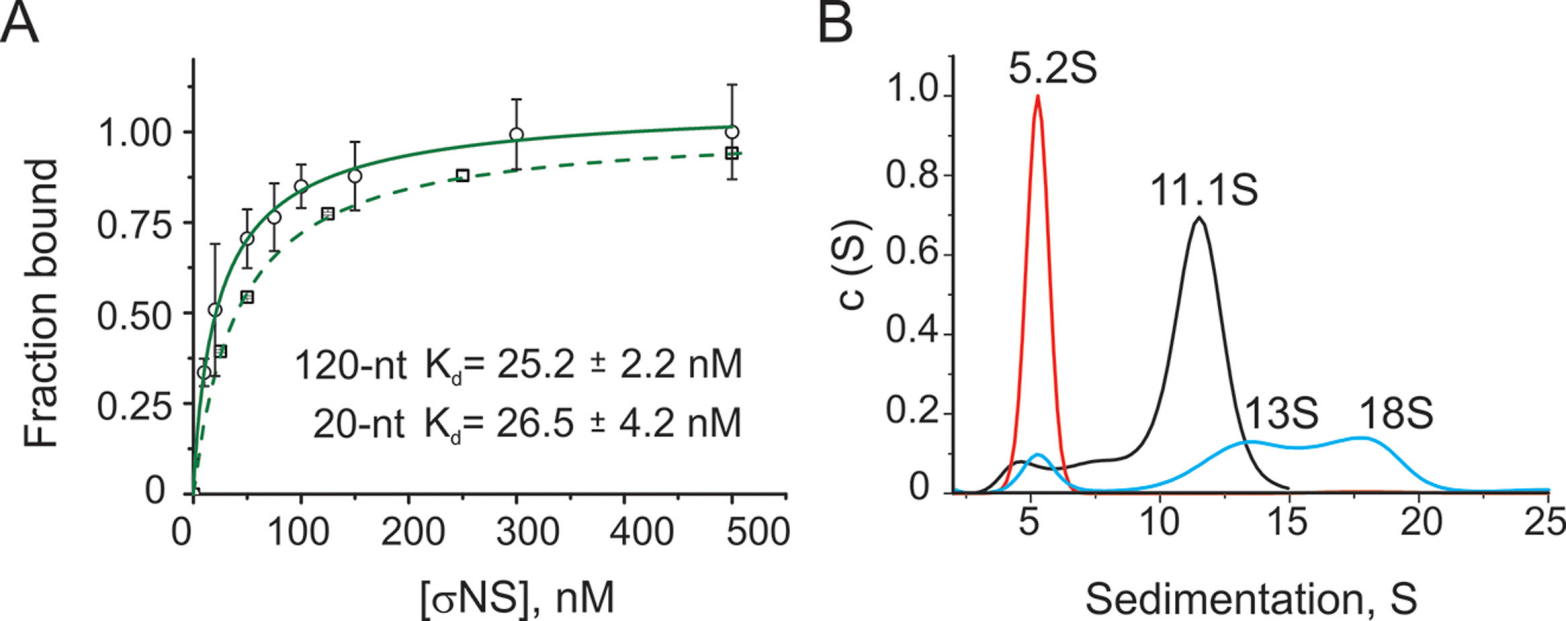
ARV σNS binding to unstructured ssRNAs. (**A**) The apparent affinities (*K*_d_) of σNS for unstructured ssRNAs, measured by fluorescence anisotropy (FA). Normalized FA have been fitted to a one-site binding model for a 120-nt long RNA (solid line) and a 20-nt long RNA (dashed line), yielding *K*_d_ values of 25 ± 2.2 and 26.5 ± 4.2 nM for a 120-mer and a 20-mer, respectively. The scale of normalized FA data for the 20-mer substrate is offset for clarity. (**B**) σNS binding to a 120-nt long RNA, analysed by sedimentation velocity (SV). A c(S) distribution plot of the RNA alone (60 nM), sedimenting at 5.2S (red), and σNS hexamers (6 μM, black). σNS-RNA ribonucleoproteins are formed upon incubation of 60 nM RNA with 6 μM σNS (shown in blue), resolving into 13S and 18S species.

ARV σNS has been previously reported to bind ssRNAs, as well as single-stranded DNA *in vitro* (14). In order to characterize σNS binding to ssRNA quantitatively, we designed several defined ssRNA substrates of variable lengths (20–120 nt), with minimal propensity to base pair (Supplementary Table S1), dye-labeled at the 5′-end for use in fluorescence anisotropy (FA) measurements, as described in ‘Materials and Methods’ section. These substrates bind σNS with high affinity, with the estimated apparent dissociation constant (*K*_d_) of 26.5 ± 4.2 nM for the 20-nt RNA (Figure 2A). Similar affinities are observed for equivalent 20-nt long ssDNAs (Supplementary Figure S5), suggesting the protein does not have any preference for ssRNA over ssDNA. The 120-nt ssRNA also binds σNS with similar affinities to those obtained for the 20-mer (Figure 2A).

Due to the oligomeric nature of the ARV σNS, it is possible that a single hexamer could bind several RNA strands simultaneously. At molar excess of the 20-mer RNA (Supplementary Table S3), native ESI-MS reveals 1:1 and 2:1 RNA:hexamer complexes, thus directly demonstrating binding of up to two RNAs per single hexamer. Other smaller protein oligomers were also detected, but only the hexamer bound RNA, suggesting that it is the functional RNA-binding oligomer. Since the stability of higher order species may be compromised in the gas phase, we also examined whether the 20-mer is capable of binding more than a single hexamer using fluorescence correlation spectroscopy (FCS). At saturating protein concentrations, the *R_h_* of the 20-mer does not increase by more than the estimated hydrodynamic size of a single hexamer (Supplementary Figure S6), consistent with binding to a single hexamer.

Given that σNS hexamer binds ssRNAs as short as 20 nucleotides, we used sedimentation analysis to examine whether a longer RNA, the 120-mer, can bind multiple hexamers. SV of the protein-free 120-mer (60 nM) reveals a single 5.2 S species for the 41-kDa RNA (Figure 2B, red). In order to be able to detect the protein component in SV experiments, we used a molar excess of σNS (6 μM) over the 120-mer, which results in formation of 13 S and 18 S RNA-containing complexes, in addition to the 11.3S RNA-free hexamer (Figure 2B, blue). We then used FCS to characterize the apparent hydrodynamic radii (*R_h_*) of these complexes. The *R_h_* of the 120-mer (∼3.6 nm), incubated with 0.5–1 μM σNS, increases to 4.8 ± 0.8 nm (Supplementary Figure S7), corresponding to the size of a single hexamer, independently measured by DLS (see above). At higher protein concentrations (5–10 μM), the *R_h_* further increases to 5.9 ± 1.0 nm, consistent with binding of a second hexamer to the RNA. Thus combining *R_h_* and *S* values of ribonucleoproteins, the estimated masses of 13S and 18S species correspond only to complexes of 1:1 and 2:1 hexamer:RNA stoichiometry, respectively. A 2:2 stoichiometry is not compatible with the sedimentation data (Table 1). Thus longer ssRNAs can bind multiple hexamers of σNS simultaneously.

**Table 1.** Hydrodynamic characterization and molecular masses of a 120-nt long RNA:σNS complexes of variable stoichiometry

Sedimenting species, S	[σNS]:[RNA] stoichiometry	Expected MW (kDa)	Calculated from experimental *S* values MW (kDa)	*δ*MW (%)	Partial specific volume (ml/g)
13	1:1	286	296	3.5	0.706
18	2:1	531	524	1.4	0.72
18	2:2	572	433	24.3	0.687

Partial specific volumes of [σNS]:[RNA] complexes were calculated based on partial specific volumes for the RNA alone (0.55 ml/g) and σNS alone (0.7321 ml/g), estimated as described in ‘Materials and Methods’ section. For 13S and 18S species partial specific volumes were calculated using the ratio of masses of RNA:protein for a given [σNS]:[RNA] stoichiometry (69). Combining the *S* values and the *R_h_* values, measured by FCS, the apparent molecular masses (MW) of 13S and 18S species were calculated, as described in (70). The calculated MW of 13S and 18S species closely match those expected for 1:1 and 2:1 σNS:RNA complexes (*δ*MW < 5%).

### σNS hexamer binding results in RNA secondary structure destabilization

**Figure 3.**
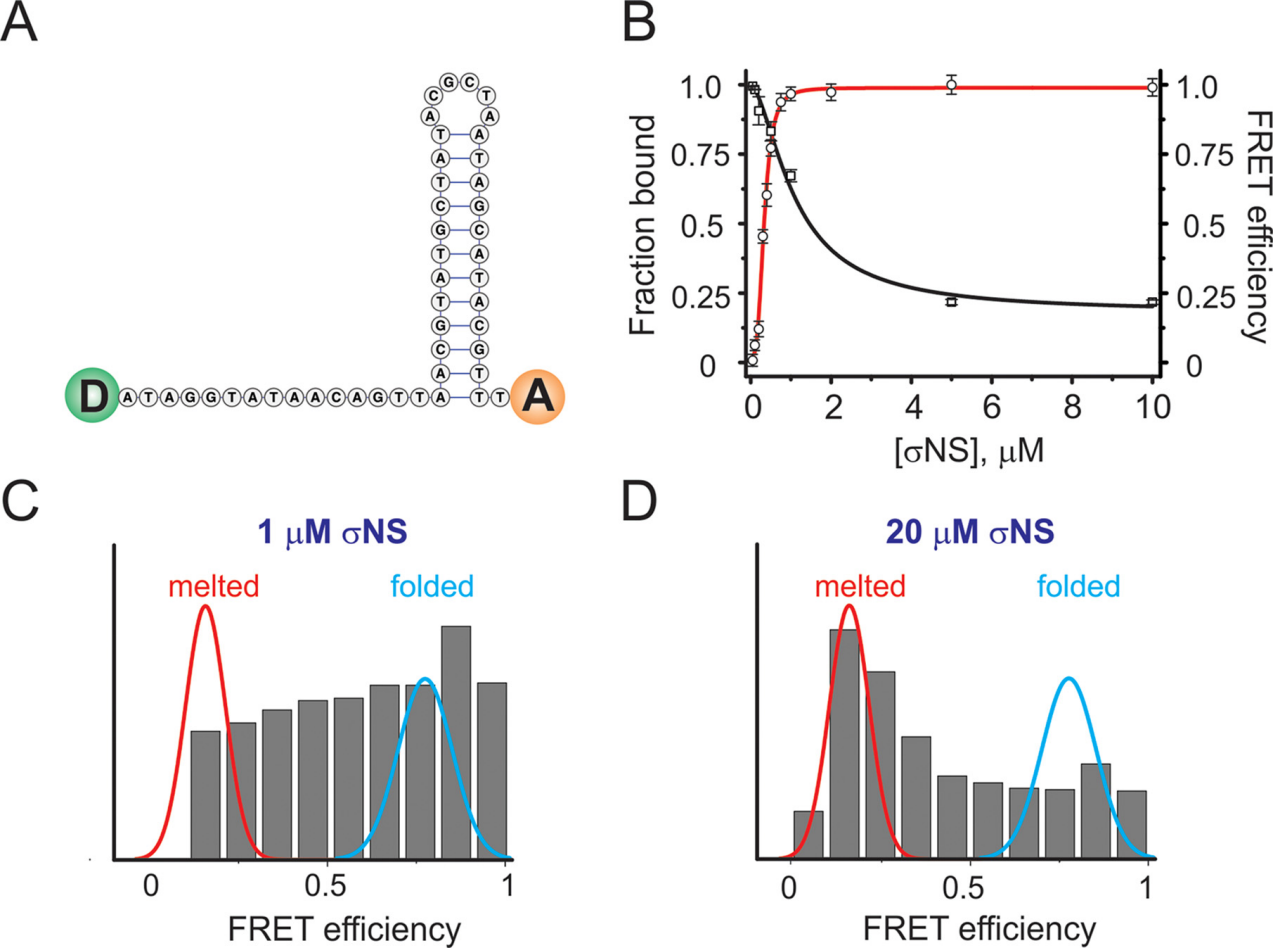
σNS binding to partially double-stranded nucleic acids results in helix-unwinding. (**A**) A minimum folding energy structure of a 46-nt long stem-loop used for helix unwinding assays. FRET donor and acceptor pair is schematically shown as green and orange circles at the 5′ and 3′ ends, respectively. (**B**) Binding of σNS to the stem-loop shown in (A), monitored by FA (red binding curve fit), with *K*_d_ of 333 ± 10 nM. The second fit (in black) shows changes in ensemble FRET upon σNS binding to the stem-loop due to helix unwinding (apparent *K*_d_ = 1.1 ± 0.2 μM). (**C**) Multiple unwound stem–loop intermediates, revealed by the wide distribution of the smFRET population histogram of the stem-loop probe (10 pM), incubated with 1 μM σNS. Gaussian fits of data for formamide-melted (red, low FRET) and heat-annealed (folded, cyan, high FRET) probes are overlaid for reference. (**D**) smFRET population histogram of the 46-nt stem-loop (as shown in C), incubated with 20 μM σNS.

We then examined how nucleic acid secondary structure affects its affinity for σNS. Since σNS exhibits similar affinities for both ssRNA and ssDNA, we designed a 46-nt long dual end-labeled ssDNA probe (see ‘Materials and Methods’ section), comprising a 30-nt stem-loop and a 16-nt long single-stranded region (Figure 3A). The choice of ssDNA over ssRNA substrate was motivated by the technical difficulties in synthesis of the equivalent dual-labeled RNA with extensive secondary structure, whilst the apparent affinities for ssRNA and ssDNA are similar (Supplementary Figure S5). The apparent affinity of σNS for the partially double-stranded 46-nt long substrate was significantly lower (*K*_d_ = 333 ± 10 nM, Figure 3B, red), than for the unstructured 20-nt long ssDNA. Analysis of the ARV σNS binding to the partially double-stranded 46-nt probe yields Hill coefficient of 3.2 ± 0.23, suggesting binding cooperativity, in contrast to the behavior with fully single-stranded substrates.

We then examined whether σNS binding to the stem–loop results in destabilization of its base-paired region by monitoring changes in FRET upon protein binding. The probe alone exhibits high FRET efficiency, consistent with its predicted secondary structure (Figure 3A). When titrating the probe with molar excess of σNS, a gradual decrease in FRET efficiency is observed, that plateaus at ∼5 μM σNS (Figure 3B, black), suggesting helix unwinding. Fitting the FRET data to a single site binding model yields a *K*_d_ of 1.1 ± 0.22 μM, i.e. significantly higher than that measured by fluorescence anisotropy. This result agrees with a model in which binding of a hexamer captures transiently opened single-stranded regions, leading to gradual helix unwinding at higher protein concentrations. In this model binding effectively competes with the duplex stability, leading to the higher apparent, FRET-derived *K*_d_ for unwound species.

Since the binding model described above predicts the presence of multiple species with different degrees of unwinding, we employed a single-molecule FRET detection approach using the alternating laser excitation (ALEX) method (41) in order to obtain the distribution of the unwound species. A high FRET population (*E* = 0.89) is observed for the probe alone (Figure 3C, blue), while unfolding by formamide resulted in complete conversion of high FRET molecules into a low FRET (*E* = 0.15) population (Figure 3C, red). Increasing the σNS concentration leads to a gradual shift from a high FRET population towards lower FRET values, with a significant broadening of the distribution at 1 μM protein concentration (Figure 3C). Further addition of σNS results in unwinding of more molecules (Figure 3D, and Supplementary Figures S9 and S10). The number of discernible populations does not change upon further increase in σNS (up to 20 μM), consistent with the presence of multiple folded and partially unfolded molecules at equilibrium.

### Long ssRNAs expand upon σNS binding

**Figure 4.**
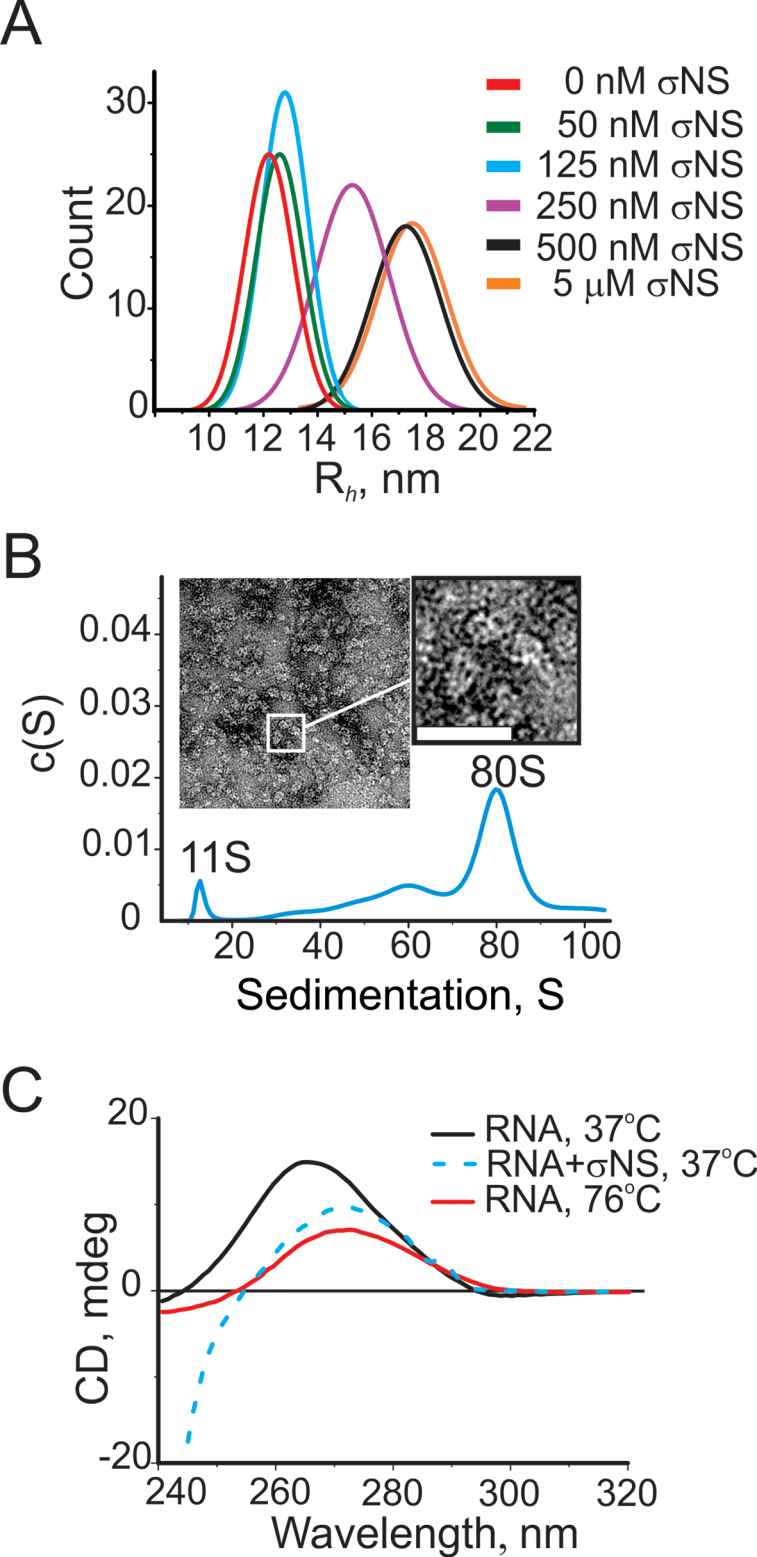
Assembly of σNS ribonucleoproteins with long ssRNAs. (**A**) Size (*R_h_*) distributions of a 3.6 kb-long ssRNA alone (1 nM, red), and upon addition of σNS, measured by FCS. (**B**) Sedimentation velocity of the long ssRNA (25 nM), incubated with σNS (10 μM). A large 80S complex with fewer smaller species is formed. Inset – negative stain EM micrograph of the σNS, bound to long ssRNAs, revealing multiple spherical ribonucleoproteins. Bar = 25 nm. (**C**) Circular dichroism (CD) spectra of the long ssRNA (as in panels A and B), reveal RNA secondary structure destabilization upon incubation with the ARV σNS. CD spectra for folded RNA alone at 37°C (15 nM, black) and thermally unfolded RNA at 76°C (red) are shown along with the CD spectrum of the RNA, incubated with σNS for 5 min (15 μM, blue dashed line). Note the significantly lower intensity of the 263 nm peak in the latter, characteristic for an A-form double-stranded helix, with its shift toward 271 nm, typical for single-stranded RNA. Below 240 nm, the CD signal from RNA is mainly dominated by the contribution of the protein.

Having examined σNS binding to short single-stranded nucleic acids with defined secondary structures, we then characterized its binding to longer ssRNAs. Given that multiple hexamers can bind ssRNAs ≥ 120 nts, it is possible that σNS binding results in RNA condensation, thus facilitating viral genome packaging (45,46). We used FCS to examine conformational behavior of the well-characterized 3.6 kb long MS2 phage genomic ssRNA (18,47,48). The apparent hydrodynamic radius of the RNA (1 nM, Figure 4A, red) does not change significantly upon addition of up to 125 nM σNS (Figure 4A, blue), while further increase in [σNS] up to 250 nM results in a marked increase in the *R_h_* of RNA (Figure 4A, magenta). The apparent *K*_d_ for binding to long ssRNA is higher (∼250 nM), than for unstructured shorter ssRNA, consistent with the high content of secondary structure in the 3.6 kb RNA (47–49). Given the high affinity of σNS for unstructured ssRNA (∼25 nM), this result implies that despite the initial binding of σNS at low nanomolar concentrations, the examined ssRNA neither aggregated nor underwent significant conformational changes. However, further addition of σNS (up to 500 nM) results in an increase of the hydrodynamic size of the RNA, which reaches saturation above ∼500 nM σNS (Figure 4A). These results suggest that the observed increase in *R_h_* reflects expansion of the RNA and formation of large ribonucleoproteins (*R_h_* ∼ 18 nm), rather than aggregation of multiple ssRNA molecules. In order to characterize the shape and dimensions of these ribonucleoproteins, we performed SV analysis of long ssRNAs (60 nM), incubated with 6 μM σNS. Multiple spherical 50–90 S complexes, presumably of variable RNA:σNS stoichiometry, are formed (Figure 4 B), consistent with the appearance of negative stain EM samples (Figure 4B, inset).

Taking into account the helix-unwinding activity of the ARV σNS, we hypothesized that the observed expansion of the long ssRNA is likely due to its conformational rearrangement, caused by destabilization of its secondary structure. We therefore examined RNA secondary structure changes upon σNS binding using circular dichroism (CD) spectroscopy. A positive peak at 263 nm was observed for RNA alone (Figure 4C, black), consistent with the largely base-paired RNA in an A-duplex conformation (48). Thermal unfolding of the RNA results in a shift of the 263 nm peak to 271 nm (Figure 4C, red), as expected for an RNA in a single-stranded form (29). Similar changes occur upon incubation of the RNA with 15 μM σNS (Figure 4C, dashed blue line), suggesting that binding of the ARV σNS induces melting of local secondary structures in large folded ssRNAs, resulting in their expansion.

### σNS facilitates RNA annealing upon helix unwinding

**Figure 5.**
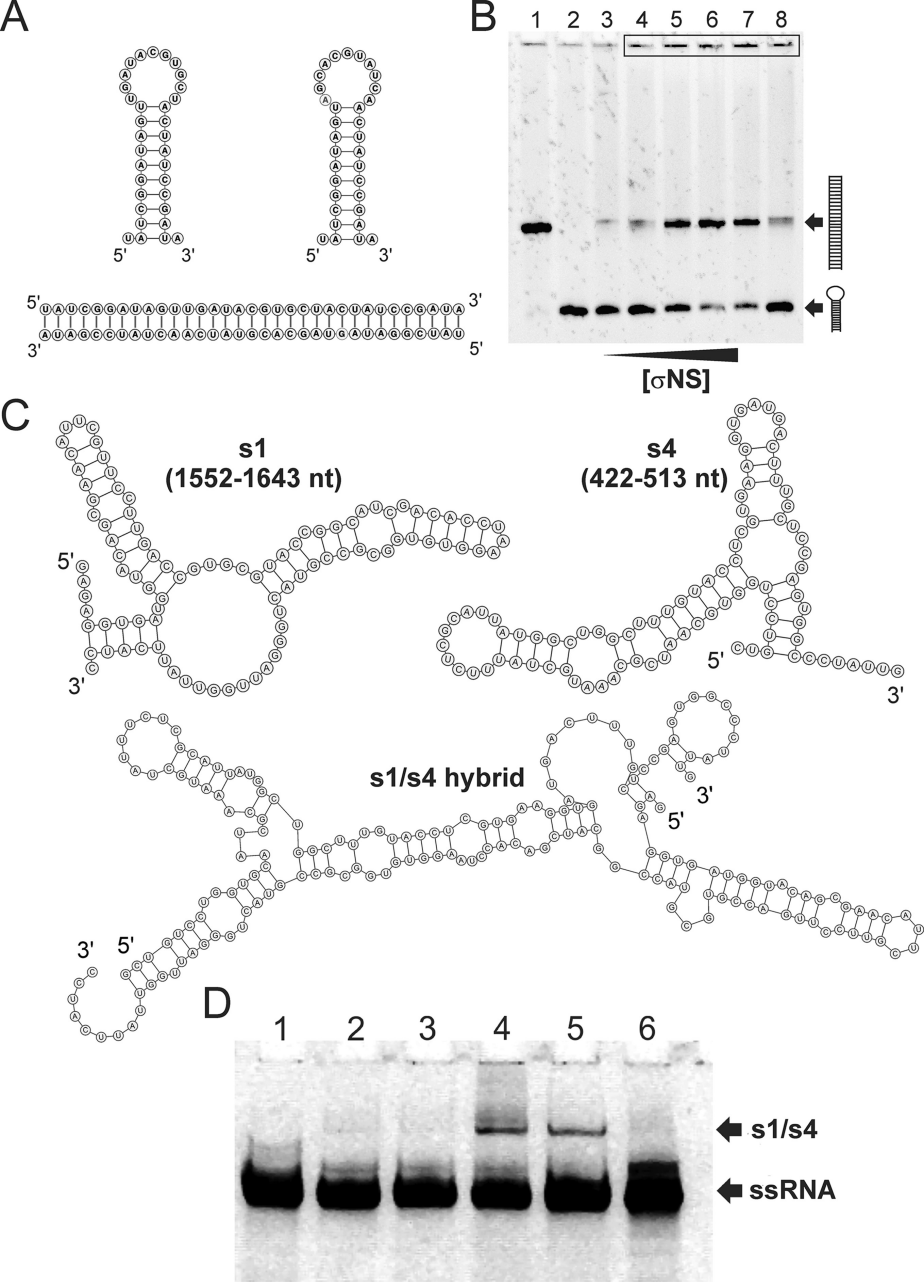
Helix destabilization facilitates strand-annealing activity of σNS. (**A**) Complementary ssRNA stem–loops used in the strand-annealing assay, as described in ‘Materials and Methods’ section (top), with the resulting re-annealed dsRNA form shown below. (**B**) RNA stem–loops (100 nM each, shown in A) were incubated with σNS at 37°C for 5 min, as described in ‘Materials and Methods’ section. The resulting products were resolved on a 10% (w/v) native PAGE, and visualised by staining with SYBR Gold. DsRNA and ssRNA stem–loops were loaded into lanes 1 and 2, respectively, while lane 3 contains the annealing reaction incubated with 20 μM BSA protein. Lane 4 – 1 μM σNS, lane 5 – 5 μM σNS, lane 6 – 10 μM σNS, lane 7 – 20 μM σNS. Note a fraction of the RNA in the presence of σNS fails to enter the gel (top, black rectangle). Prolonged incubation with σNS up to 30 min does not yield higher amounts of the double-stranded product (cf. lanes 4 and 8; 5 min and 30 min, respectively). (**C**) MFE structures of partially complementary regions of the ARV genomic precursors of segments s1 and s4 (Top). Both RNA structures are characterised by relatively high stabilities (dG > –20 kcal/mol) preventing spontaneous hybridisation *in vitro*. Below – the MFE structure of annealed s1 and s4 strands (‘s1/s4 hybrid’) (D) ARV σNS facilitates strand-annealing of viral RNA segment precursor sequences *in vitro*. 91 nt long RNAs (shown in C) were incubated alone, or in presence of the ARV σNS at 37°C for 15 min. Strand-annealing reactions were stopped and RNA products were analyzed on a native PAGE and visualized, as described in ‘Materials and Methods’ section. Lane 1 – s4 RNA, lane 2 – s1 + s4, incubated with 20 μM BSA protein, lane 3 – s4 + σNS, lane 4 – s1 + s4, incubated with σNS, lane 5 – s1 + s4, incubated with the rotaviral non-structural protein NSP2, lane 6 – s1 RNA. Positions of S1/S4 hybrids and free ssRNAs (S1 or S4) are indicated by arrows.

Since helix-unwinding activity is often associated with a strand exchange between preformed helices (15,50,51), we then examined the strand-annealing activity of the ARV σNS using two partially complementary RNA oligonucleotides. Two self-complementary stem-loops with 11 bp-long helical stems and 11 nt-long loops were designed (Figure 5A). Incubation of both strands (100 nM each) with 1 μM σNS at 37°C for 5 min results in a small amount of double-stranded (DS) product, while a large proportion of the single-stranded form remains intact (Figure 5B, lane 4). A significant increase in the amount of dsRNA is observed when using higher molar excess of σNS (5 μM or more, Figure 5B, lanes 5–7). These results suggest that strand annealing requires more than a stoichiometric amount of σNS to unwind preformed helices. Since σNS has very low affinity for dsRNA, any RNA-bound hexamers are expected to dissociate from the newly formed duplexes upon strand annealing, thus being able to bind new ssRNAs. However, prolonged incubation with σNS does not yield more DS product (Figure 5B, lane 4 cf. lane 8). As mentioned earlier, both anisotropy and FCS measurements of the 20-mer ssRNA indicate that σNS hexamers do not associate into higher order species, while native mass spectrometry suggests multiple ssRNAs can bind each hexamer. Collectively, these results suggest that strand-annealing occurs between two complementary strands bound to a single hexamer, while large ribonucleoproteins observed on a native PAGE (Figure 5B, black rectangle at the top of the gel) indicate that a fraction of the ssRNA stays bound to a hexamer.

Having established that σNS promotes strand-annealing between short complementary RNA stem-loops, we then examined whether it exerts similar effects on longer viral RNAs, e.g. ARV segment precursors. In ARVs, segment assortment and packaging signals are believed to involve terminal untranslated regions (UTRs), extending into the protein-coding regions of each segment (52,53). We therefore examined the 3′-terminal 200-nt long sequence of the ARV segment s1 to identify a possible complementary sequence in other genomic segments. A section of segment s4, capable of forming stable (>10 bp long) inter-segmental helix with the s1 RNA was identified, as described in ‘Materials and Methods’ section. The resulting 91 nt long RNAs, representing partially complementary fragments of the ARV genomic segment precursors s1 (1552–1643 nt) and s4 (422–513 nt), respectively (Figure 5 C), were used in strand-annealing assays. Similarly to shorter complementary RNA stem–loops, incubation of s1 and s4 RNA fragments in the presence of saturating amounts of σNS resulted in strand annealing, albeit with significantly lower efficiency (Figure 5 D inset, lane 4, cf. 5 B, lanes 5–7). No detectable annealed products were observed in reactions without σNS (Figure 5 D, lane 2), or when only one of the two RNA strands was incubated with σNS (Figure 5D, lane 3). Interestingly, when the ARV σNS was substituted with its functional rotaviral homologue NSP2, the annealed RNA products were also formed (Figure 5D, lane 5). These results suggest that both proteins may perform similar functions in these related viruses, and that the specificity is encoded by RNA–RNA interactions, while the protein acts in sequence-independent fashion.

## DISCUSSION

### Comparison with other non-structural RNA binding proteins within *Reoviridae* family

Non-structural ssRNA-binding protein σNS is one of the major components of viroplasms in avian reovirus-infected cells. It has been suggested that the protein is involved in the viral genome packaging (1,3). Although various roles of the ARV σNS have been proposed (14,54), including RNA-polymerase activity (55) and segment assortment (54), its mode of interaction with viral RNAs has remained poorly understood. In order to gain insight into the mechanistic details of the σNS–RNA interactions, we first determined the quaternary organization of the functional oligomeric form of the ARV σNS. Previously, the oligomeric nature of the recombinant ARV σNS was characterized by sucrose density fractionation (14). Here, using a combination of multiple techniques, we demonstrate that the protein readily assembles from σNS dimers into hexamers at low micromolar concentrations. Native ESI-MS data suggest that assembly of functional hexamers is likely to be driven by hydrophobic interactions between multiple protein dimers and that only hexamers bind RNA. Given the high protein concentration in viroplasms during late infection (4), the concentration of σNS is expected to be above low micromolar, suggesting the protein functions as a hexamer *in vivo*.

While both avian and mammalian reovirus σNS, as well as other viroplasm-forming non-structural proteins from *Reoviridae* have many common features, these proteins share very little, if any sequence homology. Non-structural ssRNA-binding proteins of most non-turreted *Reoviridae*, including bluetongue virus (BTV), rotaviruses, and some phytoreoviruses assemble into large oligomers, having a ring-like appearance (16,56–58). Interestingly, the secondary structure composition of the ARV σNS is rather similar to that of rotavirus non-structural protein NSP2, which assembles into octamers, with an estimated 35% of β-sheet. This likely reflects functional similarities between the two proteins, since β-sheet rich surfaces are often involved in nucleic acid binding (59). Likewise, viroplasm-forming non-structural proteins of some turreted reoviruses assemble into RNA-binding octamers (60), while in mammalian reoviruses (MRV) *σ*NS forms 7–9S species without obvious ring-like architecture (61), suggesting that the ring-like organization of this class of proteins is not unique amongst *Reoviridae*. It is unclear whether the viroplasm-building blocks of most turreted *Reoviridae*, e.g., ARV and MRV, are different in their quaternary organization from the non-structural viroplasm-forming proteins of non-turreted viruses. However, given a small fraction of octameric σNS species, observed in native ESI-MS spectrum at high protein concentration, it is possible that different functional oligomeric states of these proteins may be important for protein-RNA and protein-protein interactions during formation of viroplasms and subsequent virus assembly.

Both mammalian and avian reovirus σNS readily bind ssRNA, forming large ribonucleoproteins of variable stoichiometries (13,14,62). Interestingly, RNAse A treatment of large ribonucleoprotein complexes formed by MRV σNS releases 13S–19S particles (61), with similar sedimentation properties to the 13S–18S ribonucleoproteins observed when the ARV σNS hexamers were bound to 120-mer RNAs. Although MRV σNS has been shown to bind RNA with positive cooperativity and some preference for ssRNA over ssDNA (13), our results suggest ARV σNS binds RNAs lacking secondary structure without apparent cooperativity, with similar affinities for both ssRNA and ssDNA.

Previously, helix-unwinding activity has been reported for a number of non-structural proteins from *Reoviridae* family, including rotavirus NSP2 and MRV σNS (13,63). However, no strand-annealing activity has been demonstrated for these proteins so far. Both reovirus σNS and rotavirus NSP2 appear to be non-specific ssRNA-binding proteins, while BTV NS2 has been reported to preferentially bind BTV segment precursors (64). Detailed structural characterisation of both σNS and NS2 is needed to see whether these functionally related proteins play similar roles in RNA segment assortment and packaging.

### Biological implications of the RNA chaperone-like activity

Our results suggest that σNS hexamer appears to be the functional RNA-binding species. Previously, σNS has been proposed to act as a condensing agent for long viral ssRNAs in viroplasms (1). Here, we provide a direct experimental evidence that its effect on long ssRNA contrasts with charge neutralization-driven RNA compaction, or sequence-specific ssRNA collapse, previously demonstrated for some icosahedral ssRNA viruses (18). These results suggest that although the ARV σNS is capable of recruiting ssRNAs into viroplasms with high nanomolar affinity, it does not condense it, but rather causes the expansion of bound ssRNAs. This expansion reflects its helix-unwinding activity, demonstrated for both long ssRNA and ssDNA substrates.

Since hexameric σNS does not exhibit ATPase activity and ring-like shape of typical hexameric helicases (65), the observed helix-unwinding activity is likely driven by its binding affinity. The ARV σNS reportedly does not bind nucleic acid duplexes, therefore helix destabilization must involve binding of hexamers to stretches of single-stranded RNA, resulting in further recruitment of neighbouring, spontaneously melted single-stranded regions. This model is compatible with the observed broad population of partially unfolded substrates that are formed during σNS binding to the hairpin substrate.

Given its non-specific ssRNA-binding and ATP-independent helix-unwinding activity, ARV σNS exhibits typical characteristics of an RNA chaperone (15). While it appears to be indispensable for viral replication, it is not present inside virions. Therefore, σNS must dissociate from the pre-assorted RNAs prior or during encapsidation. Likewise, RNA chaperones do not require ATP for their helix-unwinding activity, and once the correct RNA structure is folded, their presence is no longer needed (66). Binding of RNA chaperones often relies on multiple electrostatic interactions with their targets (15). Similarly, RNA binding by σNS is mediated by multiple electrostatic contacts, since σNS-RNA complexes readily dissociate under high ionic strength conditions, while several arginine residues have been shown to be important for binding to RNA (14). While many RNA chaperones have intrinsically disordered regions that undergo folding when bound to RNA (15), CD and SAXS indicate that the bulk of the protein is well folded. However, *in silico* secondary structure and disorder sequence analysis using Phyre2 (67) predicts an intrinsically disordered region encompassing the first seven residues at the N-terminus, as well as few regions within the C-terminus (residues 329–338 and 361–367). Previously, it was shown that the deletion of the first 11 residues of the ARV σNS, as well as Arg365Leu substitution abrogates RNA binding (14). Interestingly, while in mammalian reovirus σNS the first N-terminal residues are also essential for RNA binding, they are predicted to form an amphipathic α-helix (61), contrasting with the secondary structure organisation of the ARV σNS.

**Figure 6.**
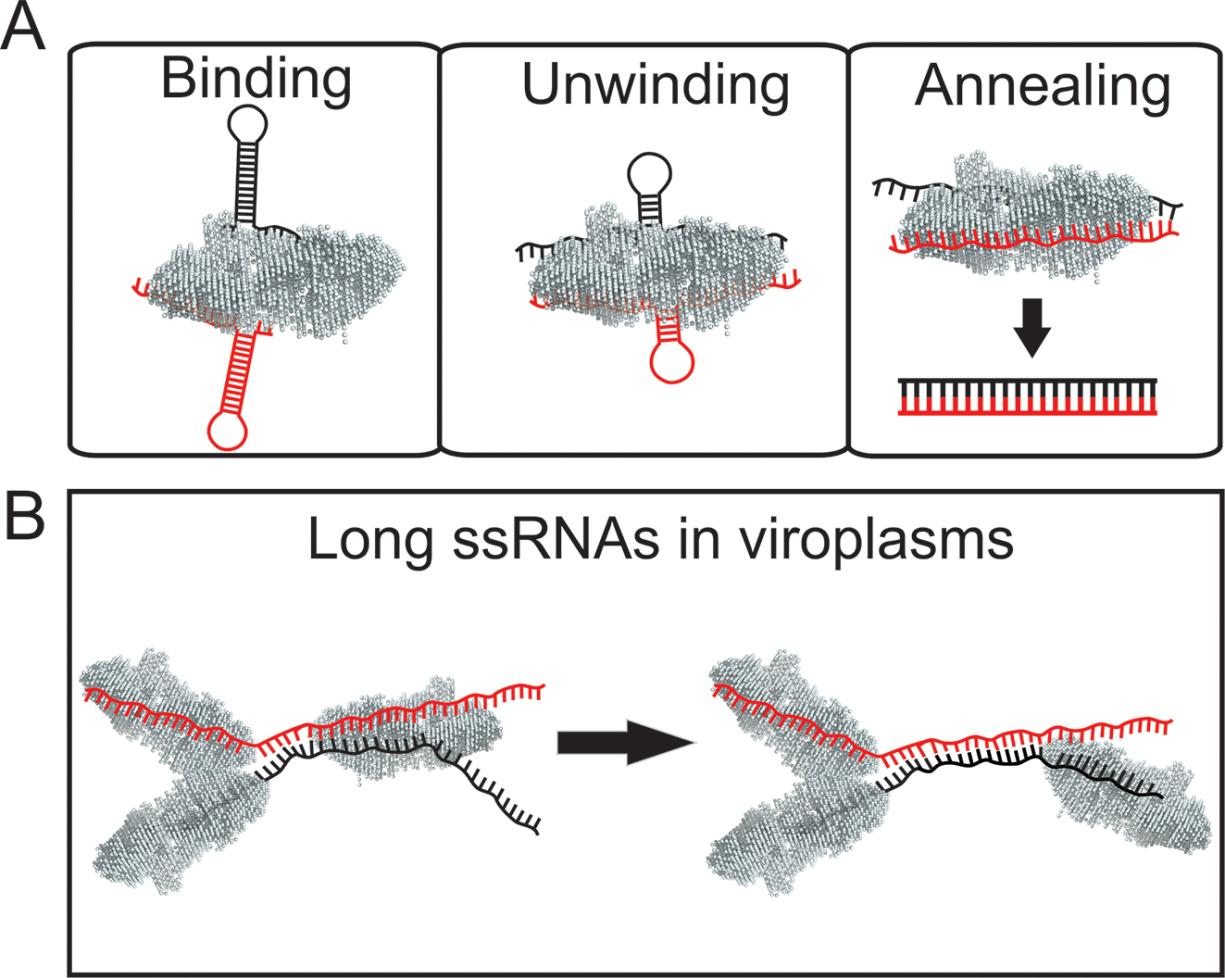
ARV σNS acts as an RNA chaperone. (**A**) *Binding mode*. σNS hexamers bind to ssRNA, including stem–loops with partial sequence complementarity (shown in red and black). Longer single-stranded stretches (>10 nts) are required for σNS binding, while shorter loops are too small to accommodate a σNS hexamer (see Supplementary Figure S8D). Multiple RNA–σNS complexes of variable stoichiometry are formed, depending on the length of the RNA substrate. *Unwinding mode*. At low micromolar and above concentrations, σNS binding to a partially double-stranded RNA results in its gradual helix unwinding. *Strand-annealing mode*. Binding of two complementary strands to a hexamer accelerates strand annealing, likely due to the molecular crowding effect. For short RNA substrates (20–40 nt), annealing occurs when two strands are bound to a single hexamer. After annealing the resulting dsRNA is released from the σNS hexamer. (**B**) Proposed roles of σNS in ARV-infected cells. The ARV σNS accumulates in cytoplasmic inclusion bodies (‘viroplasms’) in infected cells, likely by interacting with another viroplasm-forming protein μNS (1). Multiple ssRNAs (for clarity, only two strands are shown in red and black) are bound to σNS hexamers, which mediate partial unwinding of the RNAs. Two partially complementary strands bound to a hexamer can readily re-anneal, forming a more stable duplex between the two RNA strands, thus facilitating specific RNA–RNA interactions between genome segment precursors. This results in a displacement of the hexamer from the re-annealed dsRNA region and its subsequent binding to another ssRNA region elsewhere in the viroplasms.

The strand-annealing activity of σNS appears to result from helix unwinding, followed by strand hybridization, presumably mediated by the proximity of two complementary strands bound to a single hexamer (Figure 6). This model is further supported by native mass spectrometry and sedimentation analysis, which demonstrate that σNS can bind at least two RNAs, and that the assembly of a stable hexamer is required for RNA binding. Furthermore, using fragments of the ARV genomic precursors s1 and s4, we demonstrate that σNS can promote specific RNA–RNA interactions between two segments *in vitro*. The reaction appears to be more efficient with shorter, less stable RNA stem–loops, likely as the result of higher thermodynamic stabilities of longer viral RNA fragments with multiple and more stable RNA helices. This agrees well with previous observations that mammalian reovirus σNS fails to unwind a 17-bp long helix (13), while the rotaviral NSP2 was inefficient in strand-displacement assays with duplexes over 10 bp (63). Interestingly, both ARV σNS and its functional homologue rotaviral NSP2 mediate similar strand-annealing reactions, suggesting these non-structural proteins may play similar roles in segment assortment in reoviruses and rotaviruses despite lack of apparent sequence homology. Given that rotavirus NSP2 promotes strand-annealing between the ARV genomic sequences, it appears that the assortment specificity in these viruses is primarily controlled by specific RNA–RNA interactions, facilitated by the virus-encoded, but sequence-independent, RNA chaperones. Recent studies of BTV segment assortment and packaging strongly support this model, suggesting that protein-free RNA segment precursors can specifically interact *in vitro*. However, in infected cells this process requires expression of NS2 and formation of viroplasms prerequisite for RNA replication and packaging (9).

In ARVs, strand-annealing reaction leads to a stable helix formation between the two strands, resulting in displacement of the σNS hexamer from the duplex (Figure 6 A), or its subsequent binding to a neighbouring single-stranded region on longer ssRNAs (Figure 6 B). The annealing reaction appears to be thermodynamically controlled, since prolonged incubation of complementary strands with σNS does not result in the increased yield of heteroduplexes. This implies that σNS accelerates spontaneous strand hybridization events, while the extent of the annealing reaction is controlled by equilibrium between heteroduplex and intramolecular secondary structure.

While the ARV σNS has high affinity for unstructured ssRNA, the amount of protein required for its helix-unwinding and strand-annealing activities is significantly above its *K*_d_ for ssRNA. It should be noted that early morphogenesis of ARVs occurs exclusively within the viroplasm (Figure 6B), into which σNS is selectively recruited via protein-protein interactions with the major nonstructural protein μNS (6). Thus, the effective local concentration of σNS inside viroplasms is expected to be substantially higher than in cytosol. During the early virus infection, multiple (+) ssRNAs are translated in cytoplasm, and σNS may concentrate the viral ssRNAs in viroplasms and destabilize their local secondary structures, thus assisting formation of specific RNA–RNA interactions between strands during segment assortment (Figure 6 B), consistent with helix-unwinding and strand-annealing properties of the protein.

In *Reoviridae*, RNA–RNA interactions are likely to be involved in genome assortment and packaging (10). For avian and mammalian reoviruses, RNA sequences responsible for genome packaging were shown to be located close to segmental termini, encompassing untranslated regions (UTRs), and overlapping with neighbouring ORF sequences (52,53,68). Here, we demonstrate that ARV nonstructural protein σNS displays RNA chaperone activity, which augments specific RNA–RNA interactions between different segment precursors and facilitates efficient selection and assortment of multiple genome segments, destined for encapsidation.

## Supplementary Material

SUPPLEMENTARY DATA
