# Supplementary material for: Evidence that avian reovirus σNS is an RNA chaperone: implications for genome segment assortment
Source: Nucleic Acids Res. 2015 Jun 24;43(14):7044–57. doi: 10.1093/nar/gkv639 (PMC4538827; doi:10.1093/nar/gkv639)
Supplement: SUPPLEMENTARY DATA [file supp_43_14_7044__index.html]

Evidence that avian reovirus σNS is an RNA chaperone: implications for genome segment assortment — Evidence that avian reovirus σNS is an RNA chaperone: implications for genome segment assortment — SUPPLEMENTARY DATA 

# Evidence that avian reovirus σNS is an RNA chaperone: implications for genome segment assortment

## SUPPLEMENTARY DATA

- SUPPLEMENTARY DATA
