# Supplementary material for: Evidence that avian reovirus σNS is an RNA chaperone: implications for genome segment assortment
Source: Nucleic Acids Res. 2015 Jun 24;43(14):7044–57. doi: 10.1093/nar/gkv639 (PMC4538827; doi:10.1093/nar/gkv639)
Supplement: SUPPLEMENTARY DATA [file supp_gkv639_nar-00350-h-2015-File008.pdf]

SUPPORTING DATA

Supporting Table S1. Oligonucleotide sequences used throughout the study.

Name	Sequence
F_sns	CATATGGACAACACCGTGCGTGTTGGAG
R_sns	CTCGAGGTCTACGCCATCCTAGCTGG
120-nt template	TAATACGACTCACTATAGGGAGACCTATTCTCTTTTTCTCTCCTTTTCCCCCCTGTTG CTTTATTTTCCTTCTTTGCCTTTTTCTCTCTTTTTTTTTCTTTTTTCCCTTTCTGCTTTCC TCCCCTATTCTCCCCTCTT
30-nt RNA	GACCUAUUCUCUUUUUCUCUCCUUUUCCCC
20-nt RNA	GACCUAUUCUCUUUUUCUCU
46-nt	AF488-ATAGGTATAACAGTTAACGTATGCTATACGCTAATAGCATACGTTT-TxRed
33A	UAUCGGAUAGUAGCACGUAUCAACUAUCCGAUA
33B	UAUCGGAUAGUUGAUACGUGCUACUAUCCGAUA

T7 promoter sequence is underlined.

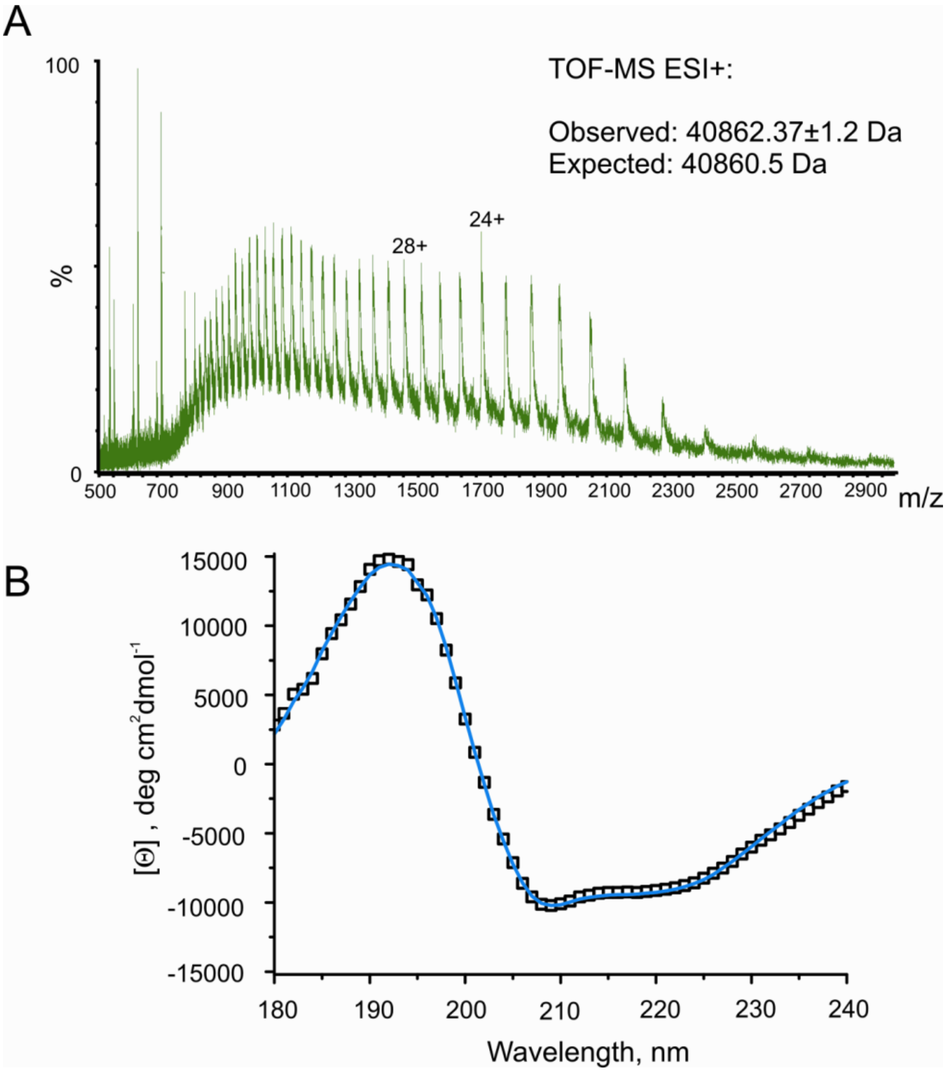

Figure S1. Characterization of the purified recombinant ARV σNS.

(A) Denaturing ESI-MS of purified σNS, the observed mass is 40862.4±1.2 Da, consistent with the expected mass of a protonated molecular ion (40861.5 Da).

(B) Circular dichroism spectrum of the ARV σNS (10 μM). Averaged spectral data from 3 scans (black squares) are shown with the best fit (blue line), corresponding to 29% α-helix, 35% β-sheet and 35% coils (see Methods for details).

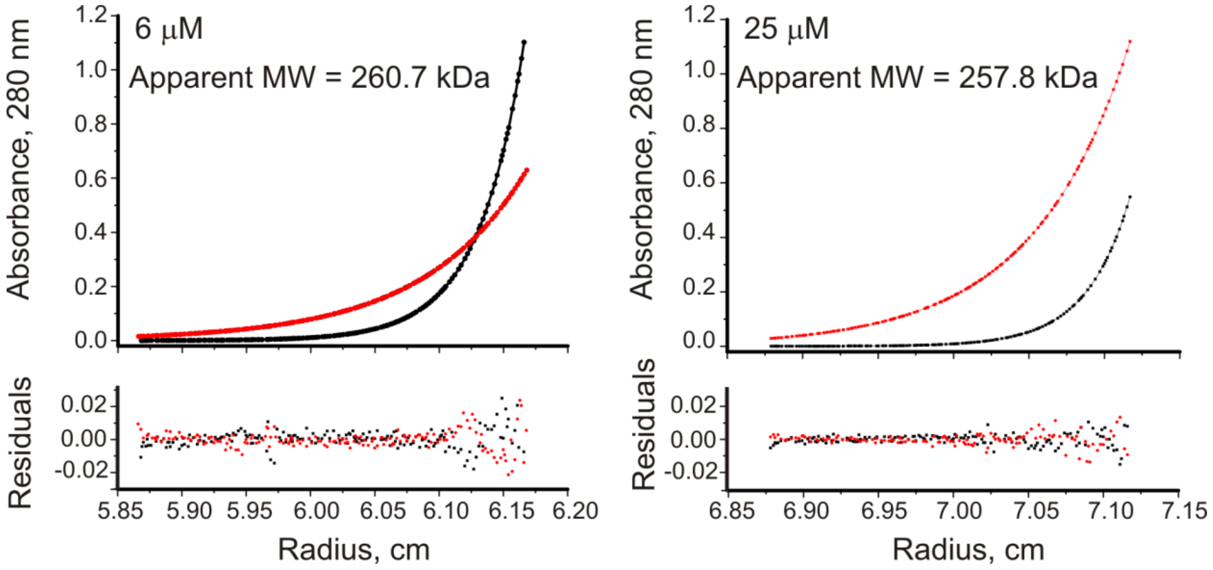

Figure S2. σNS in sedimentation equilibrium. Absorbance profiles at 280 nm at a loading concentration of (A) 6 μM (B) 25 μM at 8,000 rpm (red) and 12,000 rpm (black). Solid lines are the best-fit single species model with a molar mass of 259.3±12 kDa, with the residuals shown below.

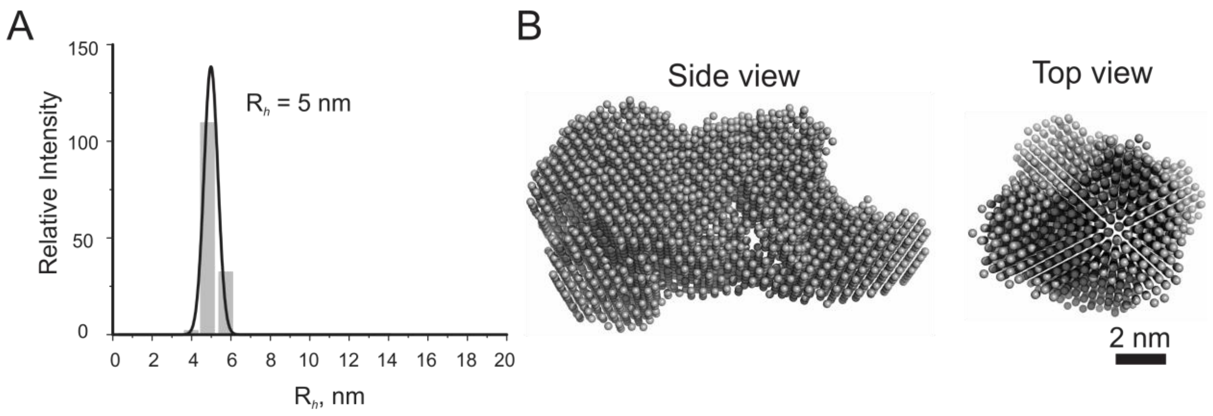

Figure S3. A. Averaged distribution of apparent hydrodynamic radii (R_h) of the ARV σNS protein oligomers (10-25 μM), measured by dynamic light scattering, as described in Materials and Methods.

B. P1 (no symmetry imposed) bead model of the ARV σNS hexamer based on SAXS data.

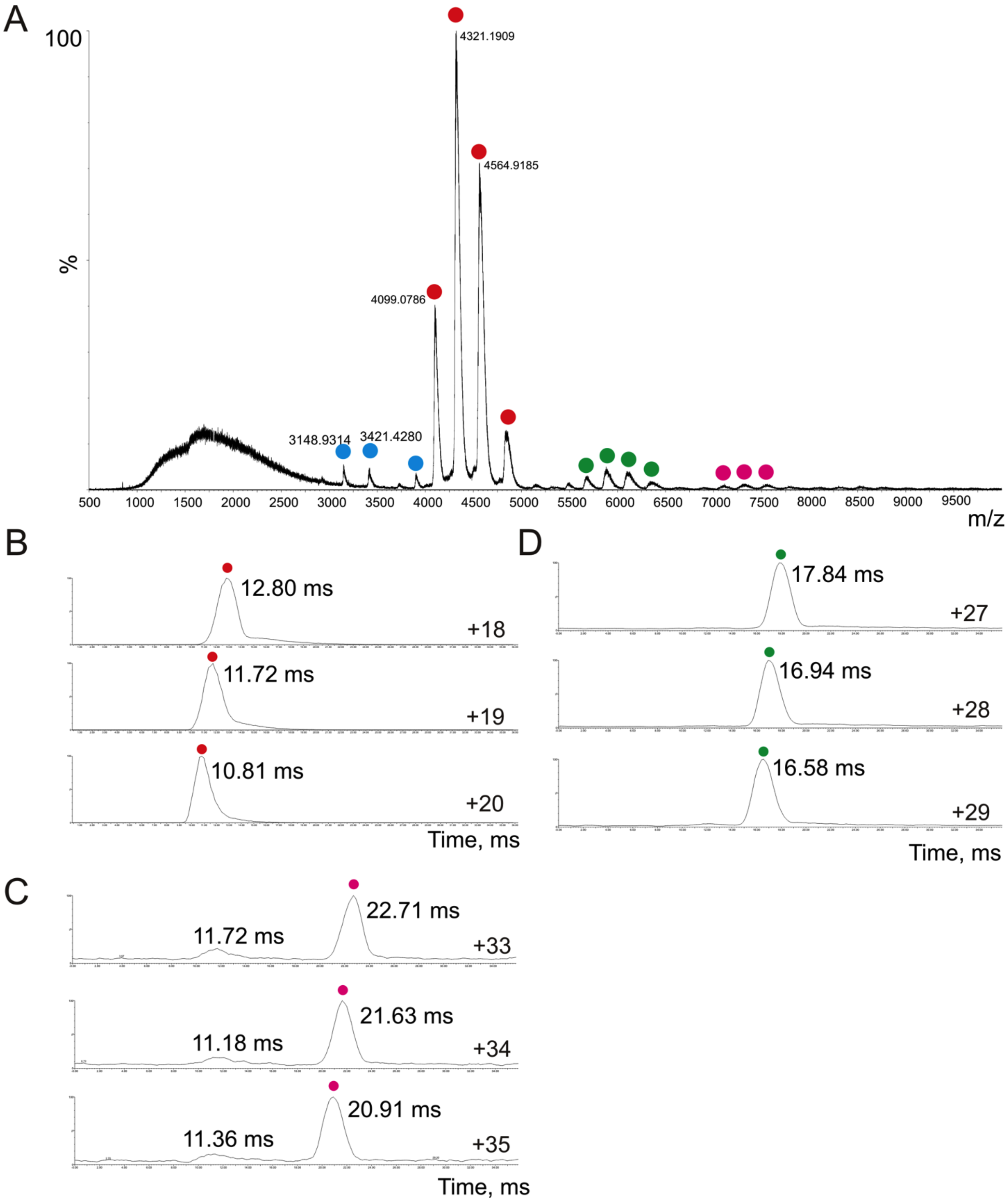

Figure S4. (A) Native electrospray ionisation mass spectrum (ESI-MS) of 25 μM σNS. Peaks corresponding to σNS monomers are labelled by blue dots, dimers are labelled in red, tetramers are shown in green, and hexamers are shown in magenta.

(B) – (D) Ion-mobility (ESI-IMS-MS) arrival time plots of σNS oligomers. Different arrival times (in ms) are shown for the 3 lowest charge states (18+ to 20+) for σNS dimers (B), 27+ to 29+ for tetramers (C) and 33+ to 35+ for hexamers (D). Summary of detected oligomeric species, measured by ESI-IMS-MS is shown in Supporting Table S2.

Supporting Table S2. Masses, cross-sectional areas, *m/z* ratios and drift time values of various σNS oligomers estimated by ESI-IMS-MS

[σNS], μM	σNS	Mass, Da	charge	*m/z*	Corrected drift time, ms	Cross-section, Å^2	Average cross-section, Å^2
12.5	dimer	81826	18	4546.89	11.80	5129.26	
		81826	19	4307.63	10.72	5283.42	
		81826	20	4092.30	9.81	5437.28	5283.32
	tetramer	163474	27	6055.59	16.82	8422.87	
		163474	28	5839.36	16.11	8638.08	
		163474	29	5638.03	15.57	8869.28	8643.41
	hexamer	246546	33	7472.09	21.68	10983.31	
		246546	34	7252.35	20.60	11169.67	
		246546	35	7045.17	20.06	11420.72	11191.23
25	hexamer	246546	33	7472.09	20.42	10816.62	
		246546	34	7252.35	19.52	11017.11	
		246546	35	7045.17	18.80	11233.09	11022.27
55	hexamer	246546	33	7472.09	20.42	10816.62	
		246546	34	7252.35	19.52	11017.11	
		246546	35	7045.17	18.80	11233.09	11022.27
110	hexamer	246546	33	7472.09	20.60	10840.89	
		246546	34	7252.35	19.70	11042.97	
		246546	35	7045.17	18.80	11233.09	11038.98

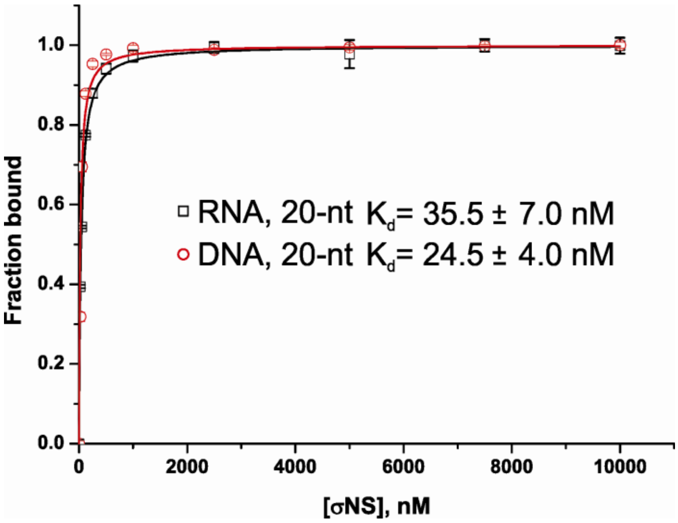

Figure S5. The apparent affinities of σNS for unstructured 20-nt ssRNAs (black), and a similar 20-nt ssDNA, measured by fluorescence anisotropy (FA). Normalized FA intensities were fitted to a one-site binding model for both substrates (solid lines), yielding K_d values of 35±7.0 and 24.5±4.0 nM for the RNA and DNA, respectively. Note, the apparent K_d of 26.5±4.2 nM, shown in Figure 2 A, was estimated independently in a separate measurement with different batches of the protein and RNA.

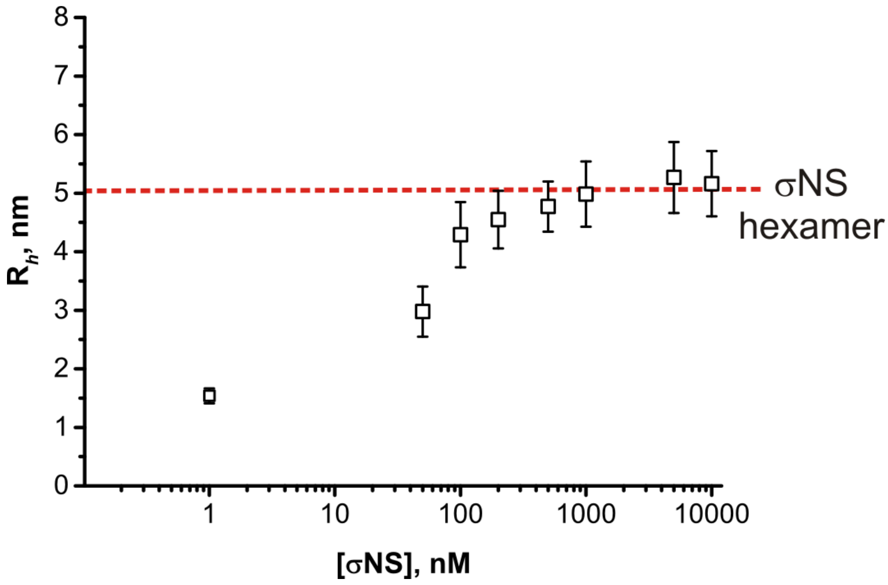

Figure S6. 20-nt long RNA binding to σNS, followed by FCS. Fluorescently labelled RNA (1 nM) was incubated with various amounts of σNS (0-10 μM). Fitting data to a one site binding model yields a K_d of 39.5±8 nM, consistent with the dissociation constant independently estimated by fluorescence anisotropy for the 20-nt long RNA (K_d ~ 25 nM). Upon saturation at 1 μM σNS, the apparent R_h does not increase beyond that expected for the σNS hexamer (red dashed line).

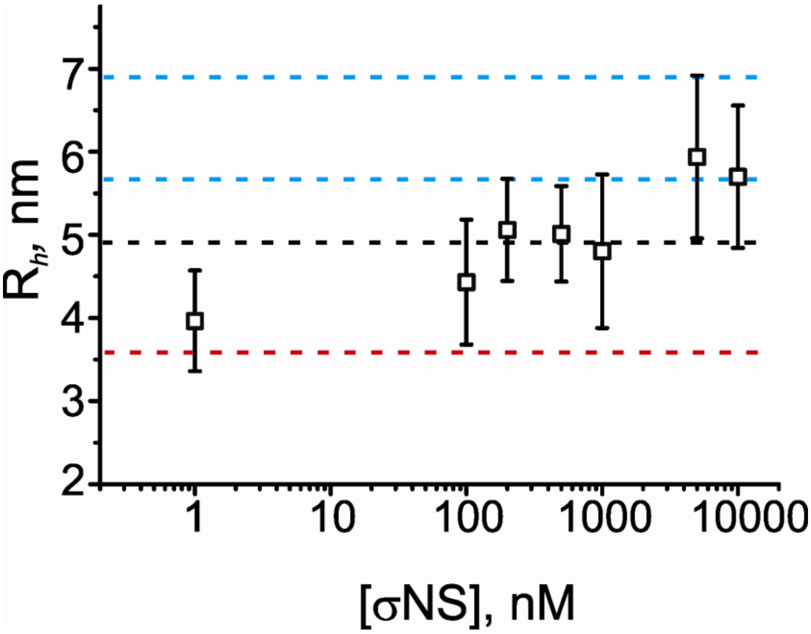

Figure S7. Apparent hydrodynamic radii (R_h) of a fluorescently labelled 120-nt RNA, measured by fluorescence correlation spectroscopy (FCS). Protein-free RNA (1 nM, red) with apparent R_h of ~ 3.6 nm, increases its size upon addition of up to 10 μM σNS. Size of the σNS hexamer is shown as black dashed line, while the blue dashed lines represent the estimated hydrodynamic radii of the 13S and 18S species, identified in the SV analysis of the corresponding RNA, incubated with 6 μM σNS (see Figure 2 B, main text).

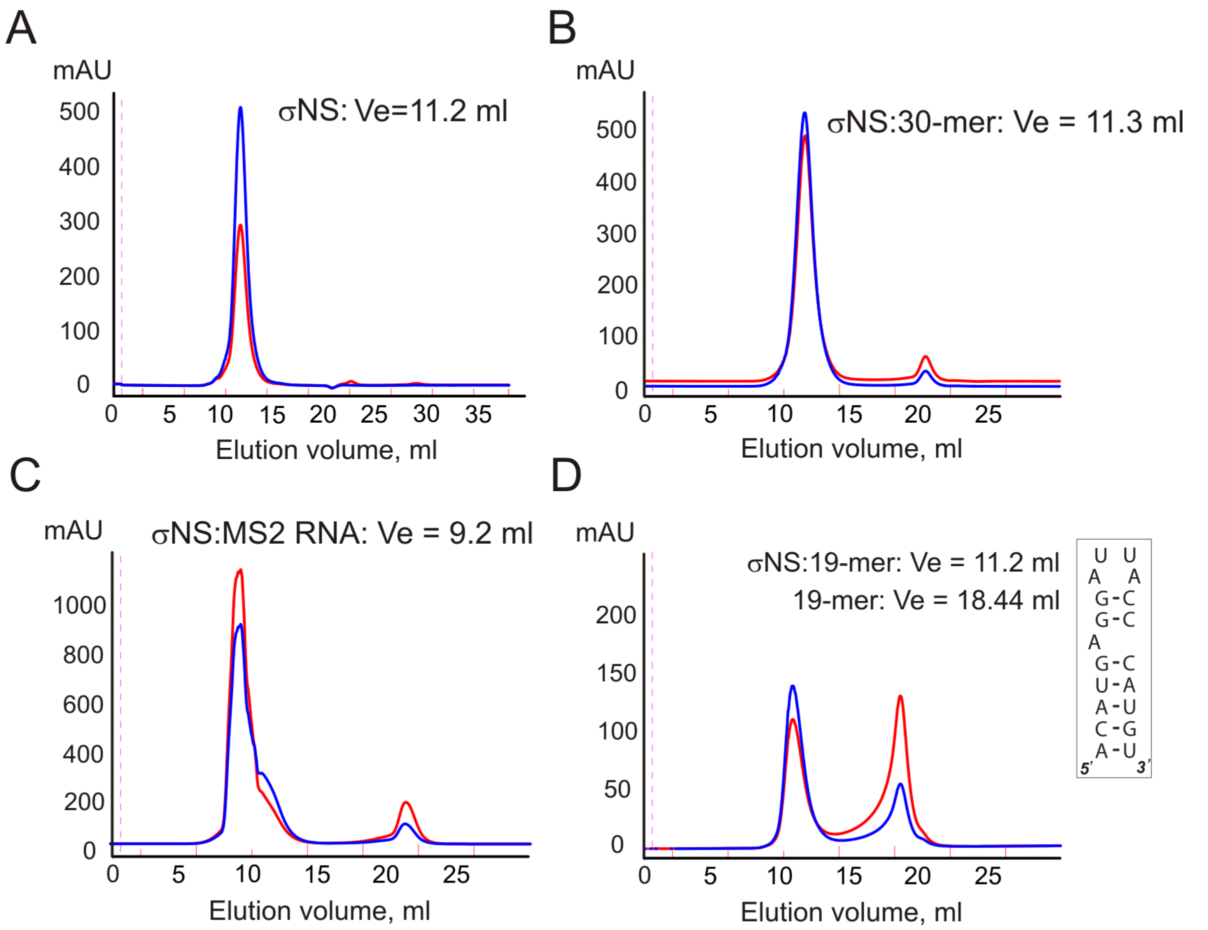

Figure S8. Size exclusion chromatogram (SEC) profiles of σNS and σNS-RNA complexes. SEC elution profiles from Superdex 200 300/GL column (equilibrated with 25 mM HEPES-Na, pH 7.8, 150 mM NaCl, at +4°C). Absorbance at 280 and 260 nm is shown in blue and red, respectively, with the injection point indicated by a dashed magenta line. Ve – elution volume (in ml). (A) 1 mg of σNS; (B) σNS:30-mer RNA complex. Note the increased 260/280 nm absorbance ratio compared to the RNA-free σNS shown in (A). RNA-free σNS and σNS-30-mer RNA complex have similar Ve, suggesting that although σNS hexamers bind short RNAs, they do not form large aggregates. (C) σNS:MS2 RNA complex. Long RNAs (3.6 kb MS2 phage RNA) bind multiple copies of σNS, forming large aggregates. (D) SEC of the 19-nt long RNA stem-loop (4-nt long loop, shown on the right), incubated with σNS (2 μM RNA:40 μM sNS). Almost no apparent RNA binding was observed, judged by only slightly altered (280/260 nm) ratios of the 11.2 ml peak, corresponding to the σNS hexamer alone (Ve=11.2 ml). As expected, a short RNA stem-loop was eluting separately (Ve = 18.44 ml), further indicating absence of σNS binding.

Supporting Table S3. Molecular masses of σNS:RNA ribonucleoproteins, detected by ESI-MS.

Detected	Observed mass, Da	Expected mass, Da	σNS:RNA stoichiometry
20-nt RNA	6140.99±0.26*	6141	N/A
σNS monomer	40859.85±0.12*	40860.5	N/A
σNS dimer	81738.23±0.75	81722	N/A
σNS tetramer	163606.75±12.28	163444	N/A
σNS hexamer	251422±25.5	252301	Hexamer : 1 RNA
σNS hexamer	257437.33±30.2	257442	Hexamer : 2 RNAs
σNS octamer #	339542.75±24.0	339162	Octamer : 2 RNAs

*Masses estimated by denaturing ESI-MS

#At higher micromolar σNS concentrations (25-150 μM) the octameric species with two RNAs bound were also observed, however, given that no octamers were seen in sedimentation analyses, it is possible that these species represent an artefact of the electrospray ionisation process at high protein concentration.

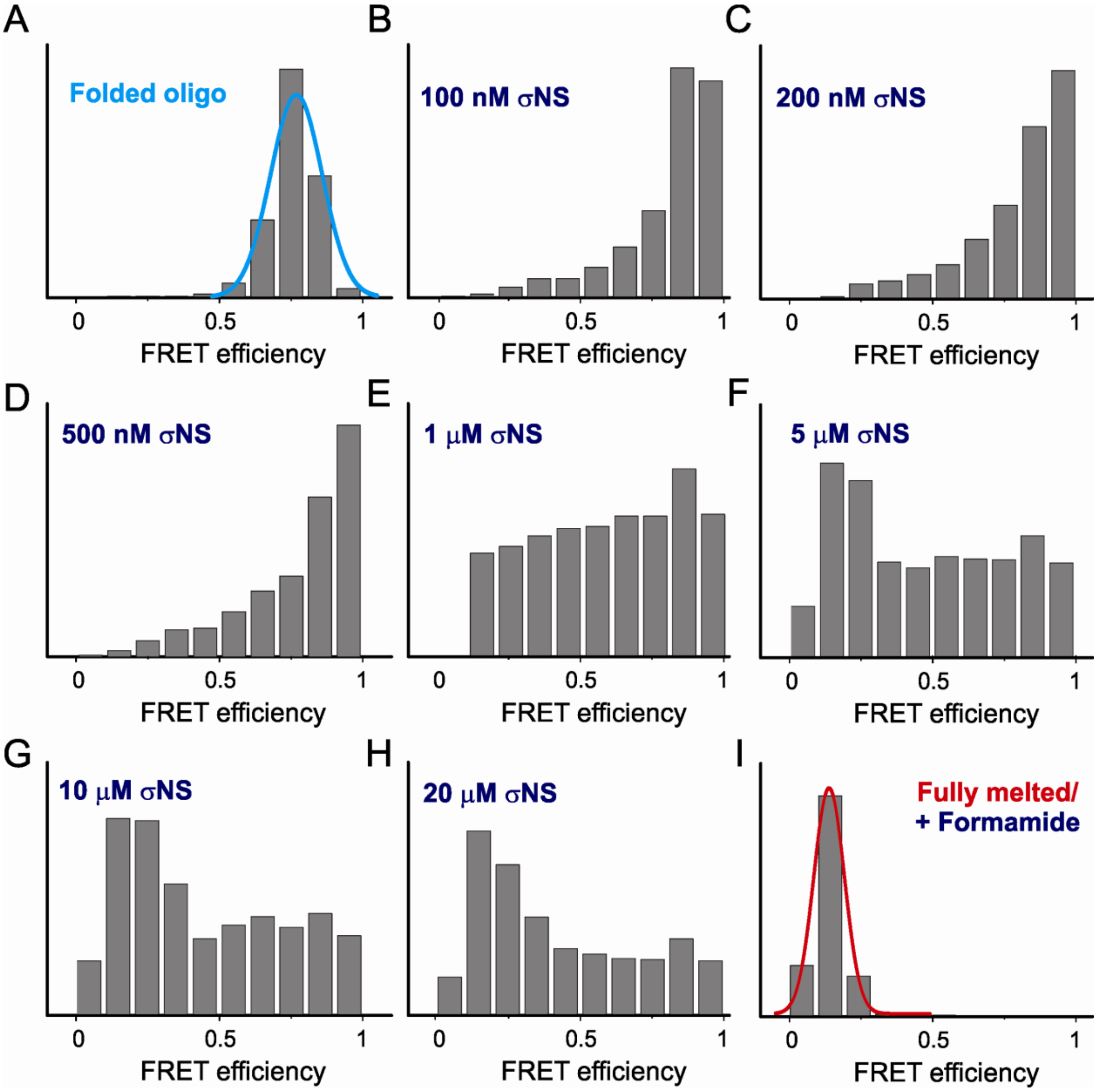

Figure S9. Population histograms obtained from ALEX smFRET measurements of the dual-labelled 46-nt stem-loop. (A) 10 pM of fluorescently dual-labelled stem-loop was measured to produce a histogram, with the overlaid Gaussian fit shown in cyan. Panels (B-H) show the histograms of the probe as in (A), incubated with increasing concentrations of the ARV σNS (100 nM – 20 µM). Note there is an apparent shift of FRET population towards higher FRET efficiency (panels B-D), caused by changes in local environment of fluorescent reporters due to initial protein binding. Panel (I) shows the data for formamide-melted probe.

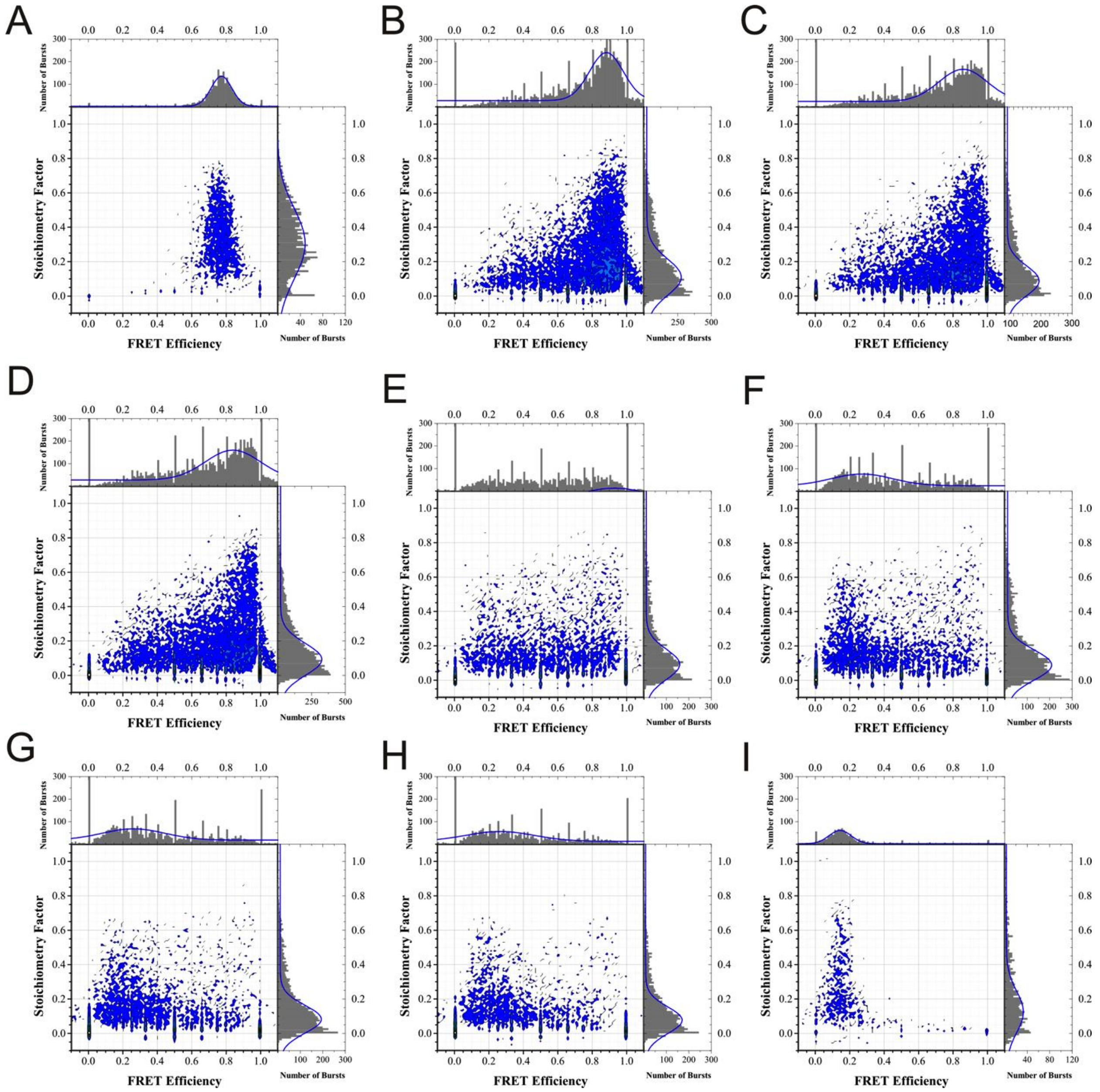

Figure S10. Two-dimensional histograms of fluorescent photon bursts, showing the ratiometric observable E (FRET efficiency) and S (stoichiometric factor), produced from Alternating Laser Excitation (ALEX) single-molecule FRET measurements of the dual-labelled 46-nt stem-loop. 10 pM of fluorescently dual-labelled folded stem-loop was measured alone (A), and with increasing concentrations of the ARV σNS (B-H), as well as in the presence of formamide (I), as described in Materials and Methods. These histograms were used to produce smFRET population histograms of the 46-nt stem-loop probe, as shown in Figures S9 and panels C and D in Figure 3.
